# Transcriptional regulation reveals potent drought tolerance mechanisms in contrasting genotypes of *Cajanus cajan* (L.) Millspaugh

**DOI:** 10.1186/s12870-025-07174-6

**Published:** 2025-10-02

**Authors:** Divya Gupta, Preetom Regon, Hans-Jörg Mai, Mayur Patel, Ranjeet S. Raje, Petra Bauer, Sanjib Kumar Panda

**Affiliations:** 1 Institute of Botany, Heinrich Heine University, 40225 Düsseldorf, Germany; 2 Plant Functional Genomics and Molecular Biology Laboratory, Central University of Rajasthan, Ajmer, Bandarsindri, 305817 Rajasthan India; 3 Department of Plant Science, Agricultural Research Organization, The Volcani Institute, 15159 Rishon LeZion, Israel; 4 Division of Genetics, ICAR-Indian Agricultural Research Institute, New Delhi, 110012 India; 5 Center of Excellence On Plant Sciences (CEPLAS), Düsseldorf, Germany

**Keywords:** *Cajanus cajan*, Terpenoids, LEA, Drought stress, WGCNA

## Abstract

Global warming severely impacts crop productivity, particularly in the Global South. Tropical pulse crops are nutritious staples and tolerant to harsh conditions, such as pigeonpea (*Cajanus cajan*). Two pigeonpea varieties have superior qualities, also with respect to abiotic stress tolerance: drought-tolerant Pusa Arhar 16 (PA16) and moderately drought-sensitive Pusa 992 (PA99). However, both are understudied at the molecular level. This study investigates molecular mechanisms of drought tolerance by investigating their responses to polyethylene glycol-induced drought. Superior drought tolerance in PA16 was characterized by enhanced shoot growth, photosynthetic characteristics and reduced oxidative stress as compared to PA992, while root length showed no significant difference between the varieties. Transcriptomic analysis identified differentially expressed genes among treatments and varieties, significantly upregulated under drought in PA16 versus PA992 with distinct patterns. For example, genes encoding terpenoid biosynthesis were up-regulated only in PA16, while those encoding LATE EMBRYOGENESIS ABUNDANT (LEA) proteins were drought-induced in both, PA16 and PA992. Functional enrichment analyses coupled with Weighted Correlation Network Analysis uncovered co-expression networks regulating drought-related pathways. Hence, the genotype and environment-specific gene regulation patterns suggest molecular and physiological mechanisms related to secondary metabolisms and LEA proteins underlying drought resilience in pigeonpea. This research offers potential targets for breeding drought-tolerant varieties of this important legume crop.

**Supplementary Information:**

The online version contains supplementary material available at 10.1186/s12870-025-07174-6.

## Highlights

Two pigeonpea (*Cajanus cajan*) varieties, drought-tolerant *Pusa Arhar 16* (PA16) and moderately drought-sensitive *Pusa 992* (PA992), were studied under polyethylene glycol (PEG)-induced drought stress.

Both varieties reacted to drought stress, whereby PA16 showed higher tolerance level than PA992. Transcriptomic analysis indicated genotype and environment effects.

Drought-responsive genes in PA16 versus PA992 were associated with secondary metabolite biosynthesis, particularly of terpenoids and flavonoids. Genes encoding LATE EMBRYOGENESIS ABUNDANT (LEA) proteins were significantly upregulated under PEG-induced drought stress in both varieties.

**Supplementary Information:**

The online version contains supplementary material available at 10.1186/s12870-025-07174-6.

## Introduction

Climate change poses a significant threat to Indian agriculture, primarily through the increasing frequency and severity of droughts. As a country heavily reliant on rain-fed farming systems, India is particularly vulnerable to prolonged dry spells, especially in central and southern regions that frequently experience recurrent water deficits [81]. Recent studies indicate that climate change will further exacerbate cropland exposure to drought in South Asia, including India, leading to substantial yield losses [45]. The Drought Atlas of India highlights that nearly two-thirds of India's land area is prone to drought, with a notable increase in its frequency, severity, and duration over recent decades—trends projected to worsen as global temperatures continue to rise [12]. To mitigate these challenges, a deeper understanding of crop-specific molecular and physiological responses to drought stress is crucial. Advances in transcriptomic and multi-omics approaches provide powerful tools to unravel complex plant responses at the molecular level, enabling the identification of key regulatory networks and stress-responsive genes. Integrating these insights into precision breeding and biotechnological interventions could lead to the development of climate-resilient crop varieties, ensuring sustainable agricultural productivity in the face of escalating environmental challenges.

Pulse legumes are essential crops that provide a significant source of protein, micronutrients, fiber, and oils, contributing to global nutritional food security and economic stability. Beyond their nutritional value, legumes play a pivotal role in sustainable agriculture Due to their ability to improve soil fertility and thrive on nutrient-poor soils. Despite these agro-ecological qualities, drought stress poses a severe threat to legume productivity. For example, 50% yield loss was observed in chickpeas under drought and heat stress [76]. Mungbean varieties also responded variably at different growth stages under drought stress [5]. To safeguard legume yields and maintain their ecological and economic benefits, it is crucial to understand their molecular, metabolic, physiological, and agronomic responses to drought [20].

Pigeonpea is a hardy legume and a vital crop in semiarid regions, renowned for its ability to thrive in harsh environmental conditions including drought [61]. Pigeonpea is an Indian staple in Many Indian cooking dishes, and India leads the world in pigeonpea production. Pigeonpea seeds are protein-rich, around 20–22%, good source of carbohydrates, fiber, minerals, and vitamins [29, 54, 55, 77]. Despite its small genome size of 833–858 Mb, pigeonpea is an understudied orphan legume, also with regard to drought responses, that needs to benefit from scientific research to explore its stress resilience mechanisms.

Comparative transcriptomic analysis, across different pigeonpea genotypes is powerful to provide profound insights into global gene expression profiles under drought stress, in this species. To date, only two studies are available. One study found that the variety CO5 is tolerant and CO1 is moderately sensitive to drought. Key drought-responsive genes were identified, including *ABI5, NF-YA7, WDR55, ANR,* and *ZF-HD6*, which were highly expressed in the tolerant genotype [51]. In another study, 111 drought-responsive genes were reported in a pigeonpea variety [77]. Yet, more studies are needed to better understand the regulatory patterns of coexpression networks and individual genes under drought.

Pusa Arhar-16 (PA16) is a drought-tolerant high-yielding, early-maturing pigeonpea variety with a dwarf growth habit, making it well-suited for high-density commercial planting. Its compact structure contributes to efficient weed suppression and enhanced productivity under optimal spacing conditions [70]. Pusa 992 (PA992) is a moderately drought sensitive early-maturing pigeonpea variety known for its high yield potential and adaptability, making it a valuable genetic resource for hybrid breeding programs [6]. PA16 resulted from the selection of single plant progeny from superior recombinants, derived from a population approach, involving diverse genotypes such as ICP 85024, ICP- 85,059, ICPL 267, ICPL 390, manak, and H-92–39, whereas PA 992 is the result of selection of 90,306. During variety evaluation for organoleptic traits, PA992 was considered as control due to its widely acceptance in market and PA16 was accepted as one of the most improved varieties [19]. These observations indicate that PA16 and PA992 have both superior qualities that can be exploited in future breeding. However, none of these qualities are studied at the genetic-molecular level, and the association of quality traits with specific gene functions remains unknown.

To address this knowledge gap, we imposed polyethylene glycol (PEG)-induced drought stress in a hydroponic system during the seedling stage, a critical period known for high drought sensitivity in pigeonpea, and conducted transcriptomic analysis. In Indian rainfed regions, pigeonpea frequently experiences early-season drought, which coincides with this developmental stage and can severely affect plant establishment and productivity [62]. Therefore, analyzing drought responses at the seedling stage provides physiologically relevant insights into early adaptation mechanisms. We addressed several key questions related to drought tolerance in pigeonpea: How do PA16 and PA992 respond at the transcriptomic level under PEG-induced drought stress? What key molecular pathways are activated in response to water deficit, and how do these differ between tolerant and sensitive varieties? Which specific genes play a crucial role in drought adaptation, and how do their expression patterns vary? Can transcriptomic insights help in identifying candidate genes for future studies on drought resilience in pigeonpea?

Our findings provide new insights into the molecular mechanisms underlying drought tolerance in pigeonpea and offer potential targets for improving resilience through breeding and genetic engineering approaches.

## Materials and methods

### Plant growth, polyethylene glycol (PEG)-induced drought stress treatment, and physiological analyses

**Fig. 1:**
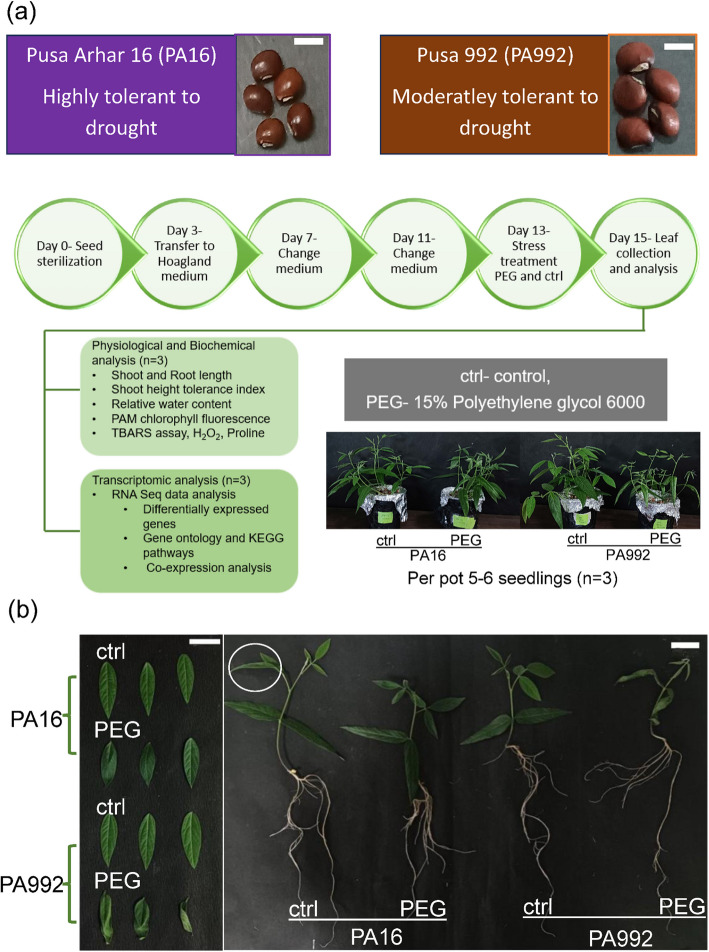
Overview of pigeonpea varieties and pipeline followed for transcriptomic analysis. **a** Upper, this illustration summarizes the characteristic features of PA16 and PA992. Lower, the flowchart explains the pipeline followed to study physiological, biochemical and transcriptomic analysis under to control (ctrl) and under PEG-induced (PEG) drought stress (Scale bar – 0.5 cm). **b** Representative images of plants exposed to control (ctrl) and under PEG-induced (PEG) drought stress. Second leaves were used for transcriptome studies (indicated by a representative circle) (Scale bar – 1 cm)

Seeds of PA16 and PA992 were procured from ICAR-Indian Agricultural Research Institute, New Delhi (28.080°N, 77.120°E) (Fig. 1a). Polyethylene glycol (PEG)-induced drought stress experiments were conducted as outlined in the flowchart (Fig. 1a). The experiments comprised three biological replicates of 5–6 plants per pot for each replicate. Briefly, seeds were sterilized in a 2% sodium hypochlorite solution for 5 min, followed by three successive washes with distilled water, each lasting 5 min. The sterilized seeds were placed on a cotton bed in Petri dishes and incubated at 28 °C for 3–4 days to promote germination. Once germinated, the seedlings were transferred to a hydroponic system containing Hoagland solution (pH 6.2) and Maintained under controlled environmental conditions, including a 14:10 light:dark cycle, 28 °C temperature, and 70% relative humidity. After 13 days of growth, drought stress was induced by treating the seedlings with 15% PEG solution for 48 h. For physiological analysis, parameters such as root length, shoot length, relative water content, Pulse Amplitude Modulation fluorometry (PAM) chlorophyll fluorescence, and shoot height tolerance index (SHTI) were measured following the method described by [52]. Additionally, oxidative stress responses were quantified, using various assays to determine the levels of reactive oxygen species (ROS) via estimating lipid peroxidation and hydrogen peroxide at the cellular level. Lipid oxidation was estimated via thiobarbituric acid reactive substances (TBARS) assay, and hydrogen peroxide via trichloroacetic acid (TCA) and potassium iodide (KI) assay. Proline as an osmoprotectant was estimated using sulfosalicylic acid, according to [52]. For gene expression analysis, leaf samples were harvested and immediately snap-frozen in liquid nitrogen and stored at −80 °C.

### RNA isolation and RNA sequencing

Gene expression analysis via RNA-seq was conducted with three biological replicates. RNA extraction, purification, and mRNA library preparation were performed by Bionivid Technology Pvt. Ltd., Bangalore, India, using the Illumina HiSeq 2000 platform. RNA was purified, and RNA integrity was assessed, with all samples exhibiting RNA Integrity Numbers (RIN) ranging from 5.2 to 7.3. Library preparation was carried out using the TruSeq Stranded Total RNA Library Prep Kit (Illumina). Sequencing on the Illumina HiSeq 2000 platform generated reads of 100–150 bp, yielding approximately 12.02 to 28.54 million reads per sample (Table S1).

### Data processing, assembly, and differential expression

Raw reads were trimmed using Trimmomatic v0.39 to eliminate adaptors and low quality base calls [7]. Quality control of the trimmed reads was performed using FastQC v0.12.0 [4] to evaluate basic statistics and confirm usability of the processed reads. Pseudoalignment of the processed reads to the reference transcriptome and further quantification of transcript abundance was performed using Kallisto v0.46.1 [8]. For reference-based assembly, the Cajanus genome from the Ensembl plant databases (https://plants.ensembl.org/Cajanus_cajan/Info/Index) has been used [77]. EdgeR v4.0 package from the Bioconductor was used for differential gene expression [57]. The *P*-value threshold was set to less than 0.05 using the Benjamini–Hochberg correction. The log2fold changes were set to ≤ −1 and ≥ 1 (corresponding to > twofold change) to consider notable differences between groups. The hierarchical clustering of differentially expressed genes (DEGs) was plotted using pheatmap v.1.0.12 [32]. Principal component analysis (PCA) was performed using prcomp v3.6.2 [63].

### Annotation

For the gene annotation, an in-house pipeline was used. Coding sequences of DEGs were retrieved from the pigeonpea database from Ensembl Plants. The protein sequences of Arabidopsis, Rice, and Pigeonpeas were downloaded from UniProt as a reference followed by a search of orthologs using the BlastP program of DIAMOND v2.18.162 with e-value 0.001 [10]. The gene descriptions like gene and protein name, Gene ontology (GO), and KEGG (Kyoto Encyclopedia of Genes and Genomes) of *Arabidopsis thaliana* were also downloaded from UniProt as reference. Further, the Blast results and gene descriptions were merged using Pandas v.2.1.4 package in Python 3.9.

### Gene set enrichment analysis (GESA)

To identify gene ontology terms linked to DEGs, a universal enrichment analysis tool “enricher” was used. *P*-value was attuned to 0.05 and the false discovery rate was 0.2 with a significant threshold. Gene ontology (GO) terms with a Minimum of 5 annotated genes were optimized to ensure robustness. The graphical representations were generated using the enrichplot v1.22.0 package of R [89]. To study metabolic pathways, the Kyoto Encyclopedia of Genes and Genomes (KEGG) database was used [27]. Protein sequences of all DEGs were retrieved from Ensembl plants and reannotation was performed using Ghost Koala in the KEGG database [28]. For the pathway mapping, KEGG gene ID (for cross reference) of *Glycine Max* (gmx), T01710 as KEGG entry number, and default cutoff score ≥ 100 were selected. Further, log2fold change as expression value of DEGs was unified into their corresponding KEGG gene ID of each sample and by using the pathview web tool, multiple state pathway map was Preferred [39].

### Coexpression network analysis

To uncover the modules that are correlated with DEGs, Weighted Gene Co-expression Network Analysis (WGCNA) analysis was performed using RNA-Seq data [35]. Pearson correlation was used to recognize co-expression similarity between DEGs. Further, to generate an adjacency Matrix, soft threshold 16 was selected followed by transformation into a topological overlap matrix to construct the network. The Minimum module size was set to 30 and mergeCutHeight was 0.25. The correlation between the traits (treatment) and module eigenvalues was assessed to identify modules of interest substantially related to the treatment. Further, interested module was explored using the string database to generate a protein–protein interaction network, further visualized in Cytoscape v3.10.0 [64, 73].

### Quantitative reverse transcription PCR

Quantitative Reverse Transcription PCR (qRT-PCR) was performed using randomly selected gene expression to validate RNA-Seq data. 100 mg of plant material was finely powdered using liquid nitrogen followed by RNA isolation using TRIZol. cDNA was synthesized using the Verso cDNA Synthesis Kit (Thermo Scientific™). Gene-specific primers were designed using Primer3Plus, with Initiation Factor 4 A (IF4-alpha) chosen as the reference gene based on its identification as the most stable housekeeping gene by geNORM and NormFinder analyses [71]. Quantitative PCR (qPCR) was performed using PowerUp™ SYBR™ Green Master Mix (Thermo Fisher Scientific) on a 7500 Fast Real-Time PCR System (Thermo Fisher Scientific). The calculation was performed using the ∆∆Ct method. The graphs were created using GraphPad Prism v8.4.0.671. The significant differences between the control and treated conditions were calculated using the unpaired t-test.

## Results

### Physiological and biochemical investigation of pigeonpea varieties under PEG-induced drought stress

To gain insight into early adaptive mechanisms of drought tolerance, we focused on the seedling stage to identify key adaptive traits in response to PEG-induced drought stress in leaves. Two varieties were compared, namely PA16 (described as tolerant) and PA992 (described as moderately sensitive), to identify mechanisms for drought tolerance through their differential responses (Fig. 1a). Growth performance was assessed through physiological parameters such as shoot length, root length, shoot height tolerance index (SHTI), relative water content (RWC), and chlorophyll fluorescence (Fv/Fm) (Fig. 1a). Oxidative stress indicators—including proline, malondialdehyde (MDA), and hydrogen peroxide (H₂O₂) levels—were measured in leaves to evaluate stress responses (Fig. 1a). Later, leaf transcriptomes were analyzed to identify potential mechanisms for stress resilience (Fig. 1a).

**Fig. 2:**
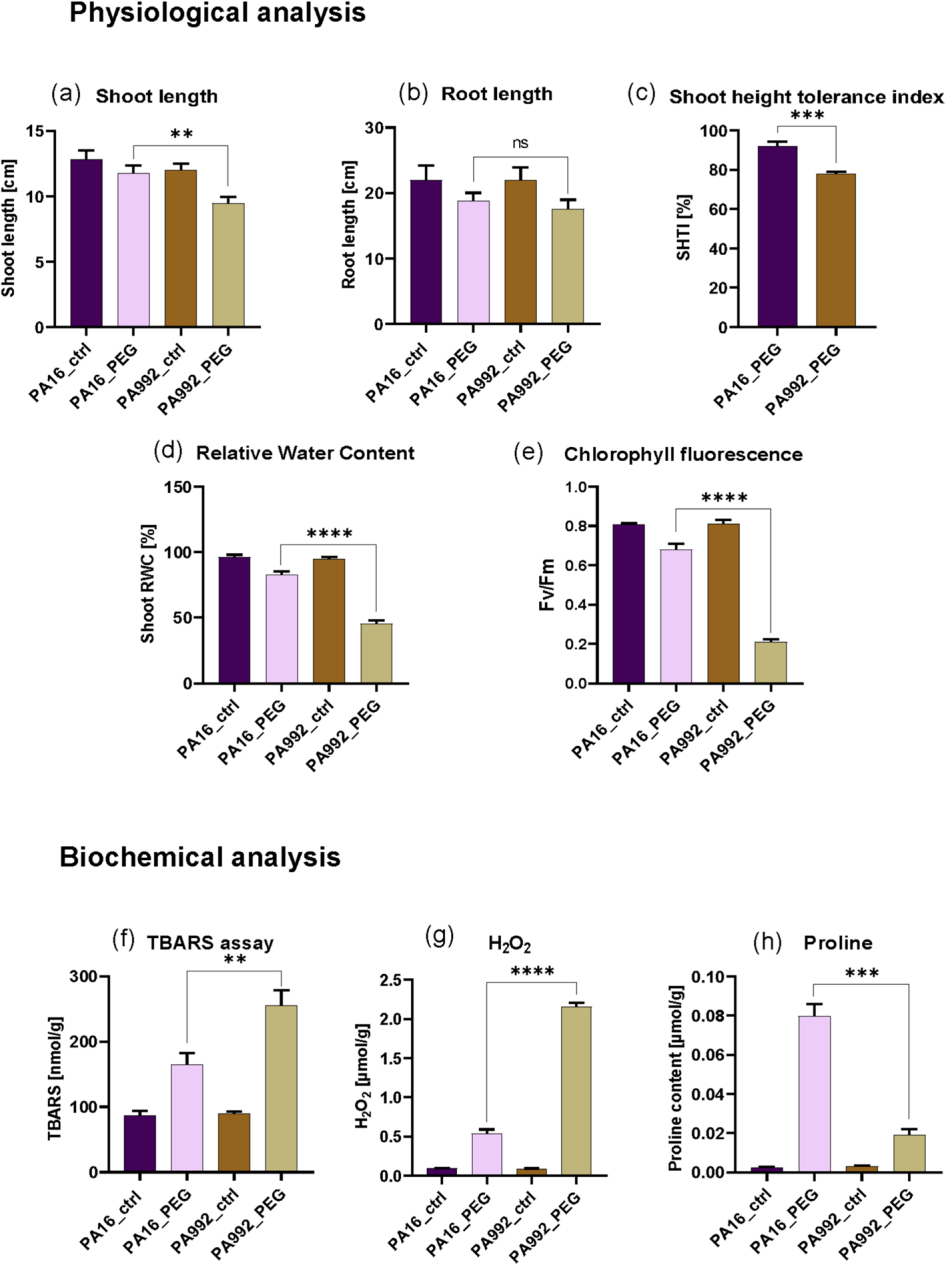
Physiological and biochemical analysis. **a** to **e**, physiological analysis, such as shoot and root length (**a**, **b**), shoot height tolerance index (SHTI) (**c**), Relative water content (RWC) (**d**), and chlorophyll fluorescence (**e**). **f** to **h**, biochemical analysis, including thiobarbituric acid reactive substances (TBARS) (**f**), H_2_O_2_ content (**g**), and proline content (**h**). The responses are compared in PA16 and PA992 under PEG-induced drought stress (PEG) versus the control (ctrl). The asterisk (*) denotes the significant difference between conditions, calculated using the unpaired t-test in the GraphPad Prism. Significant differences are displayed by *p*-values < **** 0.0001, *** 0.001, and **0.01

As anticipated, we indeed found that PA16 exhibited superior growth performance and reduced oxidative stress compared to PA992. Specifically, PA16 showed greater shoot length (Figs. 1b and 2a), while root length remained similar between varieties (Figs. 1b and 2b). Higher levels of SHTI (Fig. 2c), RWC (Fig. 2d), and Fv/Fm (Fig. 2e) were found for PA16 compared to PA992. Lower levels of TBARS reactive substances (Fig. 2f) and hydrogen peroxide (Fig. 2g) indicate reduced oxidative damage in PA16 versus PA992. Higher proline accumulation in PA16 compared with PA992 (Fig. 2h) suggests enhanced osmoprotection.

### Whole-transcriptomic analysis of PEG-induced drought stress response in pigeonpea leaves

**Fig. 3:**
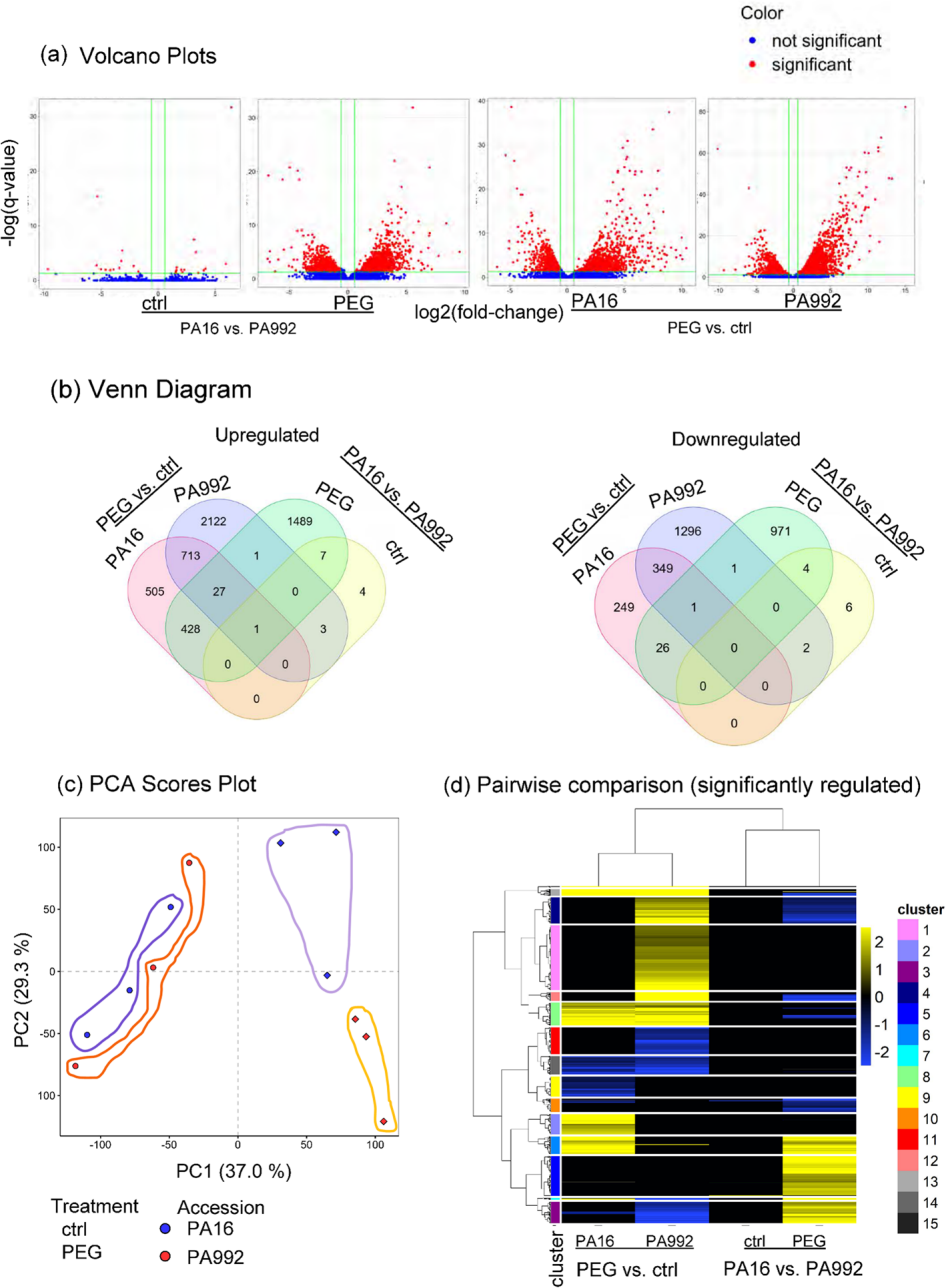
Global analysis of differentially expressed genes (DEGs). **a** Volcano plots display the expression of DEGs in the indicated comparisons. The *p-value* (Benjamini–Hochberg corrected) is adjusted to less than 10e-32. **b** Venn diagram displaying the numbers of Up and Down-regulated DEGs in the indicated comparisons. **c** Principal Component Analysis (PCA) of gene expression profiles, indicating variation in PC1 (37%) and PC2 (29.3%). Each condition is represented in triplicates. Distinct clustering of given samples is indicated by circles. **d** Hierarchical clustering, Heatmap and pairwise comparison of significantly regulated genes, as indicated. 15 clusters based on the expression of genes are shown. The color bars (yellow-blue) reflect the log2-fold change values of DEGs, indicating down- and upregulation. The responses are compared in PA16 and PA992 under PEG-induced drought stress (PEG) versus the control (ctrl) (Table S2)

Next, we conducted RNA-seq-based transcriptomics of second leaves from PA16 and PA992 PEG stress-treated and control seedlings to assess gene expression changes (Fig. 1). A total of 12.0 to 28.54 million reads with a length of 150 bp were obtained, with a GC content of approximately 45–48% (Table S1). After filtering reads and aligning to the *Cajanus cajan* genome using the Ensembl Plants database, differentially expressed genes (DEGs) were identified, with 3,018 DEGs in PA16 (control vs. treated), 5,112 DEGs in PA992 (control vs. treated), 3,702 DEGs in the treated condition (PA992 treated vs. PA16 treated) and 33 DEGs in the control condition (PA992 control vs. PA16 control) (Fig. 3a; Table S2). Hence, both varieties responded substantially to stress, while there was a significant difference in the molecular responses to PEG stress between the two varieties.

Venn diagram analysis confirmed that there were only 713 commonly upregulated DEGs (PEG-induced stress versus control) in PA16 and PA992, and 349 DEGs commonly downregulated (Fig. 3b) as compared with the uniquely differently regulated genes 505 upregulated in PA16 and 2122 in PA992, PEG-induced stress versus control, versus 249 and 1296 downregulated, respectively. The lower number of DEGs in PA16 suggests a more stable transcriptomic response under stress, which may reflect its lower susceptibility to drought compared to PA992, as previously observed in other tolerant genotypes [58]

Further, Principal Component Analysis (PCA) was conducted. PC1 explained 37% of the variance, separating control (left) and PEG-treated (right) samples, demonstrating clear transcriptomic differentiation between control and PEG treatments in both varieties (Fig. 3c). PC2 accounted for 29.3% of the variance, separating the two varieties by their PEG responses (Fig. 3c).

Finally, a heatmap combined with hierarchical clustering of all significantly differentially expressed genes illustrates the complexity of expression patterns (Fig. 3d). Two major clusters were identified. One major cluster contained genes in PA16 vs. PA992 (PEG), either less or not differentially expressed between PA16 and PA992 (Fig. 3d, upper cluster). The second major cluster, instead, contains genes which were up-regulated in the PEG stress PA16 vs. PA992 (PEG) or expressed at higher level in only PA16 but not PA992 (Fig. 3d, lower cluster).

**Fig. 4:**
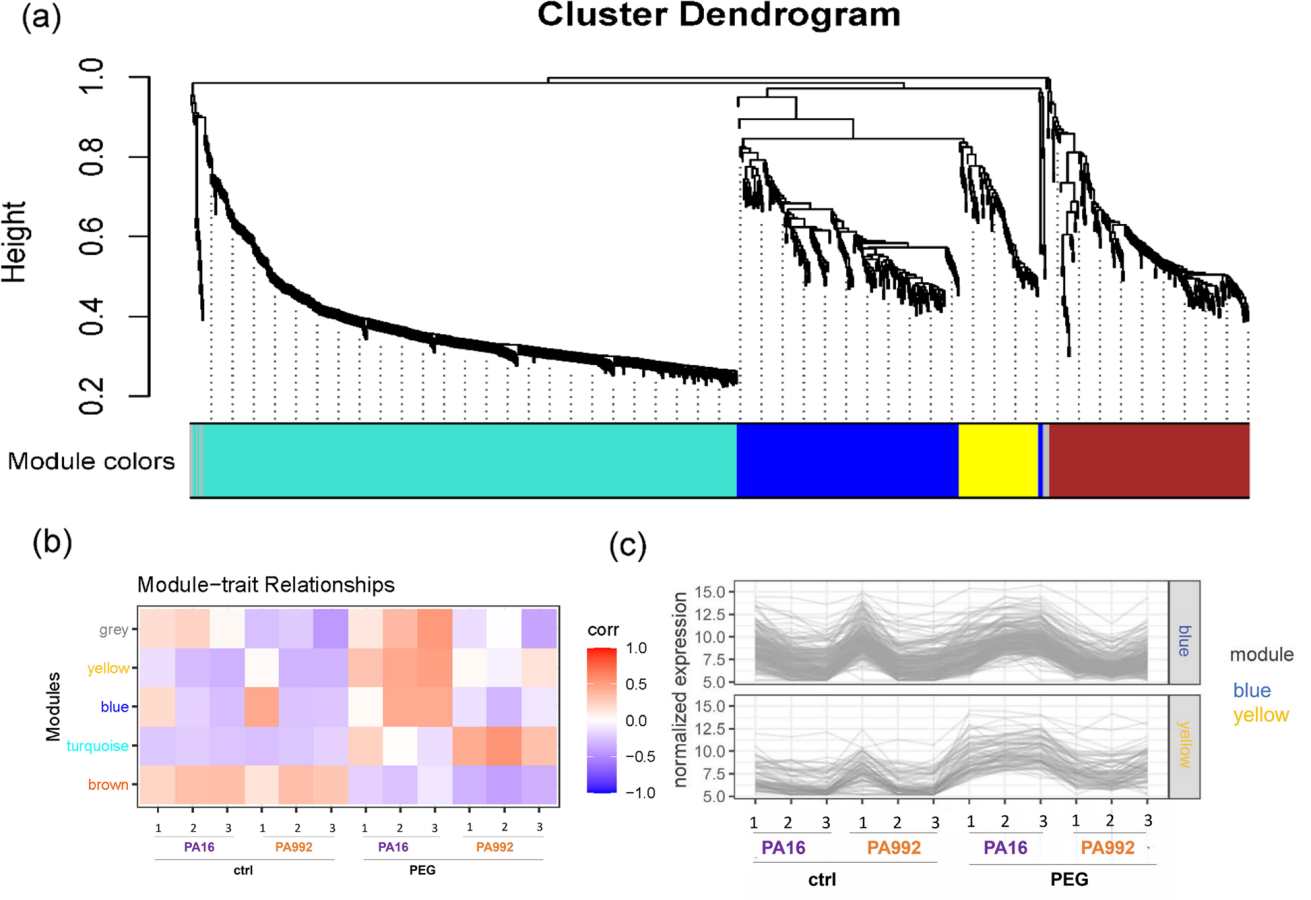
Weighted gene correlation network analysis (WGCNA) co-expression network analysis. **a** Cluster dendrogram represents different co-expression modules. The cluster dendrogram depicts distinct co-expression modules identified through hierarchical clustering, with branches representing genes grouped into specific modules based on their expression patterns (Table S3). The modules are represented by different colors. **b** Module-traits relationships represented in a heat map for the differently colored modules from (**a**). **c** Normalized expression plot of the yellow and brown module showing differentially expressed gene expression profiles. The responses are compared in PA16 and PA992 under PEG-induced drought stress (PEG) versus the control (ctrl)

To further investigate the molecular basis of drought tolerance, we used WGCNA to construct a coexpression network of differentially expressed genes in PA16 and PA992 under PEG-induced drought stress, identifying five expression modules. This analysis helps uncover gene clusters linked to drought resilience The height of the dendrogram relates to the distance metric that is used for clustering (Fig. 4a, Table S3). In dendrograms, DEGs at the top height are highly correlating with each other compared with those at the bottom height (Fig. 4a). Here, the modules depicted in grey, blue, yellow, and turquoise color showed a positive correlation with PEG treatment, with grey, blue and yellow ones particularly distinguishing the two lines by higher expression in PA16 over PA992, while the brown module displayed rather an opposite correlation and less of a distinction between the lines (Fig. 4b, c).

Clearly, there were substantial groups of genes showing similar expression patterns across the samples, which indicates that they may be coexpressed and potentially involved in similar functional pathways.

### Coexpression analysis of genes under PEG-induced drought stress in pigeonpea

Gene co-expression networks help grouping genes into functional clusters and identifying functionally relevant gene pathways. A first hint on coexpression was seen in the hierarchical clustering (Fig. 3d; Table S2). The two Major clusters further grouped into subclusters. For example, cluster 6 and 14 represent genes for secondary metabolite biosynthesis, hormone responses, and transcription factors, while cluster 8 on top of the aforementioned types of genes also contains Heat shock protein genes. Cluster 15, instead encodes LEA proteins (Fig. 3d, see highlighted frames around subclusters 6, 8, 14, and 15; Table S2).

Similarly, analysis of individual modules of a Weighted Gene Correlation Network Analysis (WGCNA) revealed that certain modules predominantly contained DEGs encoding Heat Shock Proteins (HSPs) and Late Embryogenesis Abundant (LEA) proteins (Table S3; turquoise module). The designated brown module was enriched with photosynthesis-related DEGs and some involved in the biosynthesis of secondary metabolites (Table S3). The marked blue module contained DEGs associated with lipid metabolism and stress adaptation, while the yellow module included DEGs primarily involved in the biosynthesis of secondary metabolites (Table S3). To assess the overall response to drought stress, we generated a heatmap of differentially expressed genes (DEGs) in PA16 and PA992 under PEG treatment, showing broadly consistent up- or downregulation patterns across both genotypes (Figure S1a, Table S4). These DEGs were identified by mapping pigeonpea orthologs of known drought-responsive genes in Arabidopsis thaliana, allowing functional inference based on conserved stress-regulatory roles. A focused analysis of module-assigned DEGs revealed a strong positive correlation (*r* = 0.935) between PA16 and PA992, indicating that these genes follow similar expression trends under drought conditions (Figure S1b, Table S4). Although filtered through WGCNA modules, these genes retain core drought-responsive signatures and represent highly correlated subsets that may serve as key regulators of drought adaptation in pigeonpea. The co-expression network of the yellow module was selected to visualize and highlight interactions between flavonoid and terpenoid biosynthesis-related genes, suggesting their coordinated expression under drought stress (Fig. S2). A notable finding in our study is the co-expression of genes encoding terpene synthase (TPS) with 1-deoxy-D-xylulose 5-phosphate synthase/Cloroplastos Alterados 1 (DXS3/CLA1) and squalene epoxidases (SQE), key regulators of the methylerythritol phosphate (MEP) pathway and sterol biosynthesis, respectively. Furthermore, there is coexpression of terpene synthase (TPS)-encoding genes with flavonoid biosynthesis genes coding for chalcone synthase (ATCHS), coumarate:CoA ligase (CL3), and polyketide synthase B (PKSB).

Taken together, evidence on coexpression of functionally related genes speaks in favor of regulated pathways and common transcriptional regulation mechanisms.

### Gene enrichment of differentially expressed genes in PA16 and PA992 under PEG-induced drought stress

Gene Ontology (GO) and KEGG pathway enrichment analyses provide insights into the functional implications of DEGs under drought stress. This analysis aims to determine the significantly enriched biological processes and metabolic pathways in PA16 and PA992 under PEG-induced drought stress, offering a deeper understanding of distinct stress-responsive mechanisms.

**Fig. 5:**
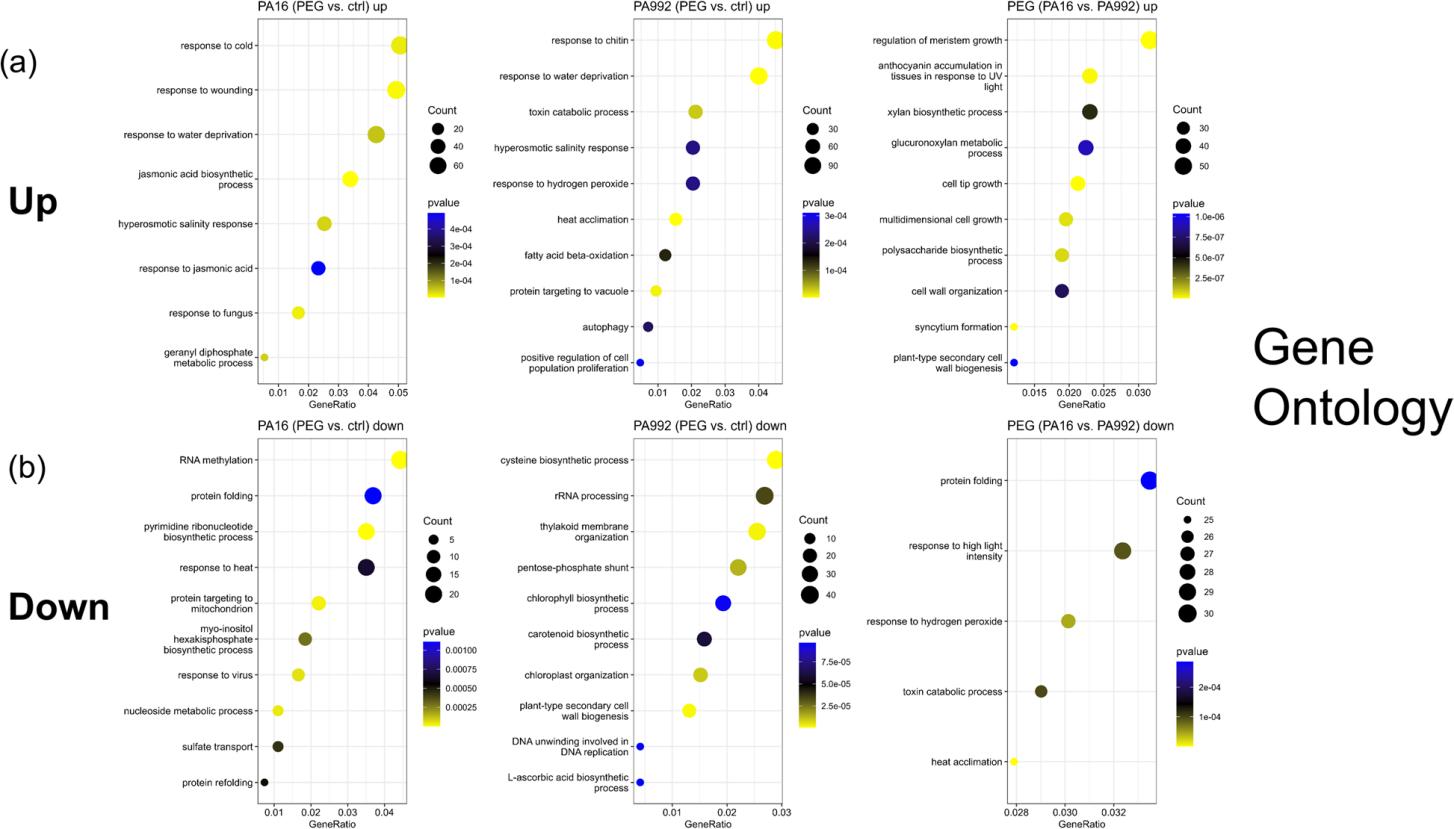
Dot plots show enriched GO terms for biological processes among the top 1000 upregulated and downregulated genes under drought stress. **a** significant GO terms for (**a**) up- and (**b**) down-regulated pathways, as indicated. The x-axis signifies the gene ratio, while the y-axis shows enriched GO terms ranked by gene count. The dot size specifies the number of genes associated with each GO term, and the dot color imitates the adjusted *p*-value (Table S4). The gradient transitions from blue, representing downregulation, to yellow, indicating upregulation. The responses are compared in PA16 and PA992 under PEG-induced drought stress (PEG) versus the control (ctrl)

GO analysis revealed distinct biological processes enriched among DEGs in both varieties. Genes upregulated in PEG treatment versus control in PA16 were enriched in response to cold (GO:0009409), response to wounding (GO:0009611), and response to water deprivation (GO:0009414) (Fig. 5a left side, Table S5), while in PA992, they were associated with response to chitin (GO:0010200), response to water deprivation (GO:0009414), and toxin catabolic process (GO:0009407) (Fig. 5a middle, Table S5). Genes higher expressed in the PEG treatment between PA16 compared to PA992 were enriched in GO categories linked to regulation of meristem growth (GO:0010075), anthocyanin accumulation in tissues in response to UV light (GO:0043481), and cell tip growth (GO:0009932) (Fig. 5a right side, Table S5). Conversely, downregulated or less expressed genes in the same comparisons were linked to RNA methylation (GO:0001510), protein folding (GO:0006457), response to heat (GO:0009408) (Fig. 5b left, Table S5), cysteine biosynthetic process (GO:0019344), RNA processing (GO:0006364), and thylakoid membrane organization (GO:0010027) (Fig. 5b middle, Table S5) or protein folding (GO:0006457), response to high light intensity (GO:0009644), and response to hydrogen peroxide (GO:0042542) (Fig. 5b right side, Table S5).

KEGG pathway analysis highlighted key metabolic pathways regulated under drought stress in both, PA16 and PA992 (Fig. S3). Commonly upregulated pathways in both varieties are glutathione metabolism, galactose metabolism, cutin, suberin, and wax biosynthesis, while commonly downregulated pathways are ribosome-related, photosynthesis, starch and sucrose metabolism, and carbon metabolism. PA16-specific upregulated pathways included fatty acid biosynthesis, ubiquinone and terpenoid quinone biosynthesis, and phagosome-related pathways (Fig. S3).

### Individual gene analysis of PEG- induced drought stress tolerance

Investigating individual gene regulation patterns can reveal mechanisms and regulatory principles or identify concrete candidate genes for future studies. Indeed, the most upregulated DEGs in PA16 include genes encoding a LEA protein (C.cajan_35494, 12.79-fold), an EC (equine renal) metallothionein family protein (C.cajan_34201, 9.8-fold), and terpene synthase 21 (C.cajan_47159, 7.9-fold) (Table S2). In PA992, the highest upregulated genes are again the same gene encoding plant EC metallothionein family protein (C.cajan_34201, 15-fold), another LEA protein, group 6 (C.cajan_10424, 12.8-fold), and a serine-rich protein-like protein (C.cajan_05970, 11.5-fold) (Table S2).

#### Differentially expressed genes in drought response: phytohormones

**Fig. 6:**
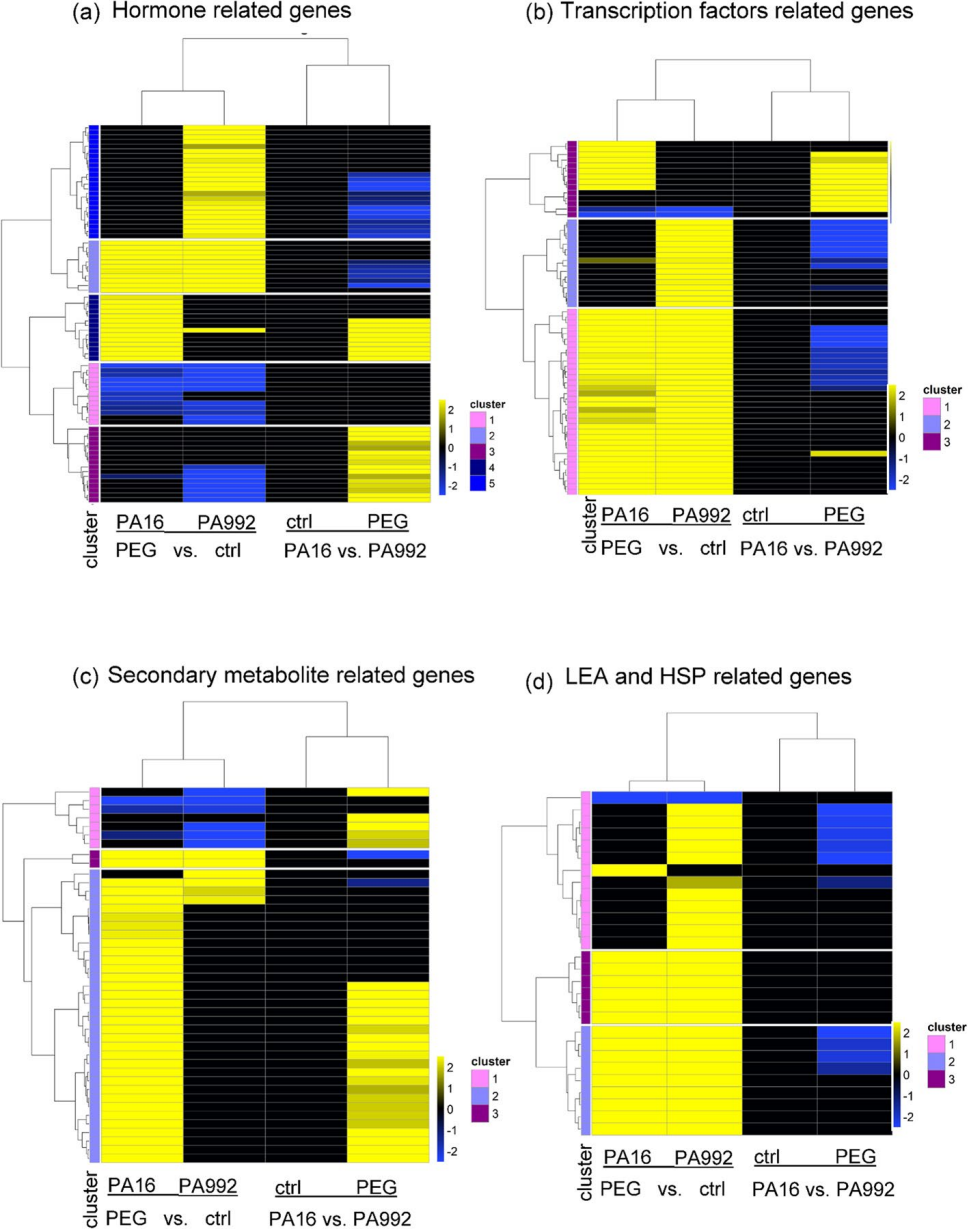
Heatmaps of the DEGs related to various pathways. **a** Hormones, **b** Transcription factors (TFs), **c** secondary metabolites, and (**d**) Heat shock protein and Late embryogenesis abundant(HSPs and LEA). The responses are compared in PA16 and PA992 under PEG-induced drought stress (PEG) versus the control (ctrl). The color bars reflect the log2-fold change values. The gradient transitions from blue to yellow, indicate down and upregulation respectively, with intermediate colors representing gradual changes in fold change values (Table S5)

The genes related to auxin, gibberellin, ABA, and jasmonate pathways were analyzed to understand their roles in stress acclimation. Differentially expressed genes were divided into five clusters based on their expression and annotation in both PA16 and PA992 varieties, highlighting their significance in drought response (Table S4, Fig. 6a). Cluster 1 represents downregulation of ABA and auxin signaling as both PA16 and PA992 exhibit reduced expression of genes encoding auxin-induced proteins, ABA receptors (PYL4/PYL6), and WAT1-related proteins, suggesting weakened ABA-dependent and auxin-mediated growth regulation. Cluster 2 exhibits upregulation of genes encoded for auxin-induced proteins, gibberellin receptor (GID1L3), and jasmonate signaling components (TIFY, Ninja-family proteins). In cluster 3 weakened Gibberellin Signaling in PA992 was observed with downregulation of genes related to GID1 receptors and GA20ox1, suggesting impaired gibberellin signaling, but no regulation of GA20ox1 was observed in PA16. Cluster 4, jasmonate and auxin signaling in PA16, strongly features upregulation of genes responsive to TIFY proteins, jasmonate O-methyltransferase, and auxin-induced proteins. Notably, in Clusters 3 and 4, significant upregulation of these genes was observed in PA16 compared to PA992 under PEG-induced stress, relative to their respective controls. Cluster 5 represents the growth maintenance strategy in PA992 where upregulation of genes responsive to GA metabolism (GA 20-oxidase, GA dioxygenases, GID receptors), auxin-related genes (*ARG, GH3, X10A*), and ABA receptor PYL8 were observed (Table S5).

#### Differentially expressed genes in drought stimuli: transcription factors

In this study, the expression of various TFs in two pigeonpea varieties, PA16 (drought-tolerant) and PA992 (moderately drought-sensitive), was analyzed to understand the molecular mechanisms underlying drought tolerance.

Expression patterns of TFs in both varieties were divided into three clusters based on their expression patterns and the identified TFs were annotated based on their known roles in stress responses (Table S4, Fig. 6b). In cluster 1, genes responsive to TFs are highly expressed in both PA16 and PA992, suggesting a conserved response mechanism. The most regulated transcription factor’s encoded genes in PEG-induced drought stress response include *MYB4* and *MYB39* (secondary metabolism and stress tolerance), *DREB1C, DREB1F*, and *DREB2D* (key drought-responsive regulators), Zinc Finger CCCH domain and C2H2-type proteins (redox balance and stress signaling), *NAC55* and *NAC72* (senescence, ABA pathways, and drought signaling), *WRKY24* and *WRKY75* (hormonal cross-talk and transcriptional activation), *ERF017* and *ERF4* (ethylene-mediated drought response), and HD-ZIP proteins (morphological adaptations under drought). In cluster 2, genes encoding TFs are predominantly expressed in PA992. Here, *ERF017, ERF114, ERF118, and ERF021*, modulate antioxidant responses, stomatal regulation, and osmotic stress adaptation, and Heat stress transcription factor B-2a and HSF24, provide drought-induced oxidative stress protection. In cluster 3, genes responsive to TFs are exclusively upregulated in PA16. The most regulated transcription factor genes include *WRKY51* (stress signaling), *bHLH135, bHLH25*, and *bHLH19* (growth and stress response), *MYB21* and *MYB4* (flavonoid biosynthesis and stress adaptation), and *HSFA2b* (heat stress tolerance). In this cluster, upregulation of PA16 vs. PA992 (PEG) was also observed.

#### Differentially expressed genes in drought response: secondary metabolites

Secondary metabolites play a crucial role in plant defense and stress adaptation, particularly under drought conditions. To investigate this, RNA-seq data from PA16 and PA992 under control and drought-stressed conditions were analyzed to identify differentially expressed genes (DEGs) involved in secondary metabolite biosynthesis. Expression patterns of these DEGs were categorized into three clusters (Table S4, Fig. 6c).

In Cluster 1, most enzymes related to flavonoids and anthocyanins were downregulated, suggesting a diminished antioxidant or pigmentation response under the tested conditions in both PA16 and PA992. Cluster 2 revealed that PA16 exhibited strong upregulation of terpenoid and flavonoid biosynthesis genes, whereas PA992 showed fewer upregulated genes. Key terpenoid-related genes, including (+)-delta-cadinene synthase, alpha-farnesene synthase, nerolidol synthase, and momilactone A synthase, were significantly upregulated, indicating their potential role in phytoalexin production and stress adaptation. Additionally, flavonoid biosynthesis genes such as chalcone synthase, leucoanthocyanidin dioxygenase, and benzoate carboxyl methyltransferase were upregulated in PA16, highlighting its enhanced antioxidant defense under drought conditions. Many of these genes were also upregulated in the PA16 vs. PA992 (PEG) comparison in both clusters.. In Cluster 3, genes for momilactone A synthase and 3'-hydroxy-N-methyl-(S)-coclaurine 4'-O-methyltransferase were upregulated in both PA16 and PA992, suggesting a conserved role in stress response.

#### Differentially expressed genes in drought response: LEA and HSPs

Heat shock proteins (HSPs), dehydration-responsive proteins, and late embryogenesis abundant (LEA) proteins play crucial roles in stress tolerance. Expression levels of stress-associated proteins were analyzed in PA16 and PA992 under control and PEG-induced drought stress conditions via categorizing them into three clusters based on their expression trend (Table S4, Fig. 6d). Cluster 1: PA992 exhibited strong upregulation of genes encoding heat shock proteins (*HSP70, HSP83*, and small HSPs) and heat stress transcription factors (*HSFB-2a, HSF24*), except for *RD22*, which was upregulated in PA16. Cluster 2: Both PA16 and PA992 upregulated genes responsive to small HSPs, phosphoproteins, and desiccation-related proteins, highlighting a shared protective mechanism involving protein folding and stress adaptation. Cluster 3: Genes encoding dehydrins, LEA proteins, and embryonic proteins (DC-8) were highly upregulated in both varieties, indicating their role in osmotic adjustment and drought resilience. No differentially expressed genes (DEGs) were significantly upregulated in the PA16 vs. PA992 (PEG) comparison within this category.

#### Differentially expressed genes in drought response: iron homeostasis-responsive genes

Iron is crucial for alleviating salinity, drought, and heavy metal stress by acting as cofactor of antioxidant enzymes like catalase (CAT), peroxidase, and SOD isoforms, which scavenge reactive oxygen species (ROS) [24]. Based on the expression patterns in both PA16, and PA992, iron homeostasis-responsive genes are divided into five clusters (Table S4, Fig. S4). In PA992 but not PA16, several iron-responsive genes were significantly upregulated, primarily within Cluster 3. These include genes for Ferritin 2 (FER2), NAC domain-containing protein 100 (NAC100), Natural Resistance-Associated Macrophage Protein 6 (NRAMP6), Nicotianamine Synthase 3 (NAS3), Ferric Reduction Oxidase 2 (FRO2), Vacuolar Iron Transporter 1 (VIT1), Yellow Stripe-Like 3 (YSL3), and Iron-Sulfur Cluster Scaffold Protein ISU1 (ISU1).

### Validation of gene expression

To validate the differential gene expression patterns obtained from RNA-Seq analysis, quantitative real-time PCR (qRT-PCR) was performed on selected genes (Fig. S5, Table S6). The selected genes encode key players in drought acclimation, including *XERO1*, low-temperature-induced *LTI65, LEA14*, Terpene Synthase (*TPS*), and α-Farnesene Synthase (AFS). The qRT-PCR analysis confirmed the RNA-Seq findings, showing consistent upregulation of *XERO1* in both PA16 and PA992 under PEG-induced drought stress (Fig. S5).

In PA992, *LTI65* and *LEA14* were strongly upregulated, which suggests a drought-response strategy in PA992 primarily focused on osmoprotection and cellular stabilization. Conversely, PA16 exhibited a stronger induction of Terpene Synthase (*TPS*) and α-Farnesene Synthase (*AFS*) compared to PA992. These genes are involved in the biosynthesis of terpenoids, which contribute to drought tolerance through antioxidant activity, membrane protection, and phytohormonal signaling.

## Discussion

Pigeonpea (*Cajanus cajan*) frequently encounters water-limited conditions during cultivation, particularly during early developmental stages, where drought becomes a major constraint to yield [62, 67]. Leaves, as the primary sites of photosynthesis and gas exchange, are highly susceptible to water loss and are among the first organs to exhibit stress responses [43, 87, 88]. To simulate drought under controlled conditions, polyethylene glycol (PEG) is widely used as an osmotic agent that effectively mimics water deficit stress without causing ionic toxicity [33]. In this study, PEG-induced drought stress (hereafter referred to as drought stress) was applied to investigate the physiological and molecular responses of two contrasting pigeonpea genotypes—*Pusa Arhar 16* (PA16), a drought-tolerant variety, and *Pusa 992* (PA992), a moderately drought-sensitive one (Fig. 1a). Through integrative physiological and transcriptomic analysis, PA16 was found to outperform PA992 under drought stress, exhibiting reduced oxidative damage and higher proline accumulation. Differential gene expression analysis revealed distinct transcriptional responses between the two genotypes. In PA16, genes involved in secondary metabolite biosynthesis, particularly terpenoid and flavonoid pathways, were upregulated and likely contributed to enhanced drought resilience. In contrast, both genotypes exhibited upregulation of common stress-responsive genes, including Late Embryogenesis Abundant (LEA) proteins, indicating the activation of conserved drought response mechanisms. These findings provide insight into the molecular basis of drought tolerance in pigeonpea and highlight candidate genes and pathways that can be targeted in future crop improvement efforts.

### PEG-induced drought stress reveals varied physiological and biochemical responses in PA16 and PA992

Our study relied on PEG application to induce drought stress, and our observed physiological and molecular responses were very similar to those expected under a drought treatment. Drought stress typically reduces Relative Water Content (RWC), reflecting decreased cellular hydration, though some genotypes retain higher RWC, indicating better stress adaptation. PSII photochemical efficiency (Fv/Fm) serves as a reliable indicator of photo-oxidative damage and photosynthetic performance under drought [85]. Under drought stress, plants usually experience an increase in reactive oxygen species (ROS), particularly hydrogen peroxide (H₂O₂), which induces oxidative damage to DNA, proteins, and lipids. H₂O₂ promotes lipid peroxidation, compromising membrane integrity [44], and leading to elevated levels of thiobarbituric acid reactive substances (TBARS)—key markers of oxidative stress and membrane damage [34]. To counteract this, plants accumulate proline, a multifunctional osmoprotectant and ROS scavenger that stabilizes proteins and membranes, maintains osmotic balance, and mitigates oxidative stress [30]. Similar oxidative stress takes place in pigeonpea plants exposed to drought in our study. Biochemical analyses in the drought-tolerant pigeonpea variety PA16 revealed higher proline accumulation compared to PA992, contributing to osmotic adjustment and cellular protection. This was accompanied by lower levels of lipid peroxidation and H₂O₂, indicating effective oxidative stress mitigation. These findings are in agreement with earlier reports of elevated proline levels in drought-tolerant pigeonpea genotypes [74]. Moreover, transgenic studies overexpressing the Cold and Drought Regulatory gene (*CcCDR*) in pigeonpea and the Hydroxy-proline-Rich Protein-Encoding gene (*CcHyPRP*) in rice demonstrated enhanced proline accumulation and reduced malondialdehyde (MDA) or TBARS levels under drought stress [42], underscoring the importance of osmoprotectants and antioxidant defenses in drought tolerance. Under the applied PEG-induced drought stress, PA16 and PA992 both showed typical drought responses, similar to the ones just described. This indicates the validity of using the PEG-induced drought regime.

PA16 exhibited superior physiological growth traits compared to PA992, including higher shoot length, shoot height tolerance index, RWC, and Fv/Fm ratio—signifying better water retention and photosynthetic efficiency, a hallmark of drought tolerance. Interestingly, while PA16 showed no significant root growth under stress, it maintained shoot development, suggesting a shift in resource allocation to sustain above-ground biomass. This trend corresponds with previous findings in pigeonpea and common bean under rainfed conditions [53, 66]. Drought stress can inhibit both root and shoot growth in pigeonpea seedlings, and certain genotypes show varied responses. For instance, the pigeonpea genotype *SKNP 1004* showed an increase in shoot length along with sustained root length and dry weight when grown under rainfed (drought) conditions, indicating a high level of drought tolerance [69]. In contrast, many other pigeonpea genotypes exhibit a decline in both root and shoot growth under similar stress conditions, reflecting their lower drought tolerance [50]. The differences between these previous studies and our study can be explained by genotypic variation in drought responses as well as differences in the experimental settings.

The enhanced oxidative stress tolerance and better growth observed in PA16 compared to PA992 suggest that reduced oxidative damage may directly support improved plant performance. PA16 likely activates specific regulatory mechanisms for ROS detoxification, as reflected by the upregulation of transcription factors such as MYB15 and WRKY51, which are known to regulate lignin biosynthesis, ion homeostasis, photosynthetic efficiency, and proline accumulation [31, 41]. These findings indicate a proactive stress response in PA16 that could be validated through functional and physiological studies. Supporting this, module–trait correlation and GO enrichment analyses revealed contrasting responses between genotypes (Table S7). The turquoise module, enriched in response to stimulus (GO:0050896), response to stress (GO:0006950), and oxidative stress response (GO:0006979), correlated positively with MDA and H₂O₂ in PA992, but not in PA16, indicating higher oxidative stress in the sensitive genotype. The brown module, related to transmembrane transport (GO:0055085), ion transport (GO:0006811), and nitrate assimilation (GO:0042128), was negatively correlated with proline and RWC in both genotypes, suggesting reduced nutrient and solute movement under drought. The blue module, enriched in lipid metabolic processes (GO:0006629), showed a positive correlation with RWC in PA16 and negative in PA992, pointing to a role in membrane protection. The yellow module, enriched in terpenoid biosynthesis (GO:0016114), diterpenoid biosynthesis (GO:0016102), and lipid biosynthesis (GO:0008610, GO:0044255), was positively correlated with RWC and proline, and negatively with MDA in PA16, but not in PA992, highlighting its role in metabolite-driven drought tolerance.

### Transcriptomic insights into hormonal regulation reveals key role of jasmonate and gibberellins signaling under PEG-induced drought stress

Drought stress disrupts plant growth and development, triggering a complex hormonal response in which JA, GA, and auxin play distinct but interconnected roles in stress adaptation [16, 78]). The identification of key differentially expressed genes (DEGs) related to auxin (IAA), abscisic acid (ABA), gibberellins (GA), and jasmonates (JA) can provide significant insights into the hormonal crosstalk involved in stress resilience.

Studies have shown that gain-of-function mutations or overexpression of *GA20ox1* and *GA20ox2*, which result in increased gibberellin production, reduce water loss by limiting canopy size without affecting stomatal closure, while gibberellin-insensitive dwarf (*gid1*) mutants maintain higher leaf water content under drought conditions [26, 65]. In our study, the pigeonpea genotype PA992 exhibited signs of weakened GA signaling, evidenced by the downregulation of *GID1* and *GA20ox1* genes under PEG-induced drought stress. This suppression of GA-related genes may reflect an adaptive mechanism in PA992, potentially contributing to reduced growth and transpiration as a means to conserve water under stress.

TIFY proteins are key regulators of plant responses to abiotic and biotic stresses. The TIFY family is divided into four subfamilies based on their domain structure: TIFY, ZML (ZIM/ZIM-like), PPD (PEAPOD), and JAZ (jasmonate–ZIM-domain) [90]. Several *TIFY* genes showed significant upregulation under drought, salt, and ABA treatments [38]. In our study, the upregulation of JAZ genes under PEG-induced drought stress, particularly in PA16, indicates a dynamic role of jasmonic acid (JA) signaling in drought adaptation. Although JAZ proteins are classically seen as repressors of JA-responsive transcription factors like MYC2, their accumulation under stress does not necessarily contradict JA activation. Instead, it may reflect a tightly regulated feedback loop aimed at fine-tuning the JA response to avoid excessive defense-related growth inhibition [15].

Auxin is essential for plant growth, development, and stress adaptation. Among auxin-responsive genes, SMALL AUXIN UP RNAs (SAURs) are rapidly induced and play key roles in auxin signaling. Overexpression and silencing of *AtSAUR32* in Arabidopsis have demonstrated its involvement in drought tolerance via regulating ion leakage and quantum yield of photosystem II, both ABA-dependent and independent pathways. The upregulation of auxin-responsive genes in both PA16 and PA992 in our study suggests a general drought tolerance mechanism, based on auxin-related factors, aligning with findings in Arabidopsis [23].

The differential expression of auxin, JA, and GA related genes in PA16 and PA992 highlights a coordinated hormonal response under drought stress. Upregulation of SAURs and JAZ genes in PA16 suggests auxin–JA crosstalk, where auxin may modulate JA signaling to balance growth and defense [82]. In contrast, the downregulation of *GA20ox1* and *GID1* in PA992, alongside auxin-responsive gene induction, points to auxin–GA antagonism, potentially limiting growth to conserve water [83]. These interactions underline auxin’s central role in integrating stress and growth signals for drought adaptation.

### Transcriptional regulation of stress-responsive genes by key transcription factors under PEG-induced drought stress

Under drought stress, TF families like bHLH, MYB, NAC, DREB1, and WRKY activate gene networks involved in osmoprotection, ROS detoxification, ABA signaling, and cell wall remodeling. DREB1 binds dehydration-responsive elements to regulate stress-inducible genes, while NAC and MYB contribute to lignin biosynthesis [2, 11, 92]. WRKYs are widely involved in abiotic stress responses such as PEG6000, salt, cold, or heat stress [56, 84]. Our results correspond with previous research emphasizing the role of bHLH and MYB TFs in drought stress adaptation. The observed overexpression of bHLH135 and MYB39 is consistent with studies in Arabidopsis, where these TFs were linked to root development and suberin deposition [13, 36]. Additionally, the identification of bHLH25 and its potential interaction with JA and TIFY signaling presents a novel angle, warranting further investigation into cross-talk between hormones and transcription factors [14]. The upregulation of NAC72 and NAC55 in PA16 and PA992 in comparison to control under PEG-induced drought condition, supports previous findings on NAC-dependent regulation of stomatal function, reinforcing the role of ROS accumulation in drought-induced stomatal closure [21, 40]. DREB1 is involved in enhancing drought tolerance in several studies [51, 68, 94] supporting DREB1C/1F upregulation expression in both pigeonpea varieties. Similarly, the upregulation of WRKY24 and WRKY70 in our dataset aligns with research on wheat, where these TFs contributed to enhanced drought resilience [22, 89].

### Transcriptional regulation of terpenoids and flavonoids in mediating PEG-induced drought stress tolerance in PA16

Secondary metabolites, particularly terpenoids and flavonoids, play a crucial role in this regulatory network by enhancing plant defense mechanisms and stress adaptation, and also have antioxidant properties [3, 47, 86]. In Salvia plants, terpenoids were identified as key modulators of ROS scavenging systems under drought stress [46]. Overexpression of terpenoid biosynthesis genes in *Glycine max* (soybean) has been shown to enhance root growth and nodulation, which are critical for drought adaptation [1]. Similarly in *Pinus elliottii* the upregulation of terpenoid biosynthesis genes with enhanced defense mechanisms under drought stress [91]. Transcriptomic studies in berries and sugarcane have also shown increased production of monoterpenes and flavonoids as a protective response to drought [48, 60]. Hence, the upregulation of terpenoid biosynthesis genes in PA16 versus PA992 under drought may be a mechanism for explaining the enhanced drought tolerance in PA16. Furthermore, this finding also coincides with earlier studies showing that flavonoid biosynthesis genes are upregulated in drought-stressed buckwheat and the drought-tolerant pigeonpea variety CO5 [25, 51]. The coexpression of terpenoid and flavonoid biosynthesis genes highlights a potential cross-talk between terpenoid and flavonoid biosynthesis in mitigating drought-induced oxidative stress [60]. These findings suggest that secondary metabolites may play a dual role in stress tolerance in PA16, specifically, by enhancing ROS scavenging and stabilizing cellular structures, potentially leading to improved drought resilience.

Although our transcriptomic data do not reveal clear upregulation of specific transcription factors directly linked to terpenoid or flavonoid biosynthesis in PA16, the differential expression of genes involved in these pathways suggests the possibility of alternative regulatory mechanisms. One such mechanism is post-translational modification (PTM) of transcription factors or biosynthetic enzymes, which can rapidly modulate activity, stability, or localization without altering transcript levels [93]. Similarly, PTMs can regulate terpene synthases and prenyltransferases, thus fine-tuning terpenoid output in response to stress signals. Therefore, the enhanced production of these metabolites in PA16 may be mediated through PTM-based regulation, even in the absence of strong transcriptional cues.

### Transcriptional regulation of LEA encoded proteins revealed as conserved mechanism in both varieties in response to drought

Late embryogenesis abundant (*LEA*) gene family and heat shock proteins (HSPs) responsive genes in PEG-induced drought stress regulation, reinforces their crucial involvement in plant adaptation mechanisms by contributing to cellular stability and oxidative stress mitigation to improve drought resilience. Dehydrins (LEA Group II), have been reported to facilitate drought adaptation and seed maturation [51, 72]. The upregulation of the dehydrin-responsive gene (*XERO1*) in both PA992 and PA16 supports existing evidence that LEA proteins play a role in stress responses in general without being particularly relevant for the greater drought tolerance in PA16 versus PA992. Additionally, overexpression of dehydrin family members, *CcCDR,* and *GmDNH9* in pigeonpea and arabidopsis respectively, improved drought tolerance in transgenic lines, are consistent with our findings [18, 74]. Similarly, the upregulation of *ECP63*, LEA group I, in PA992 and PA16 corresponds with the improved drought resilience observed in castor bean *RcECP63* overexpression lines [81]. Overexpression of *LEA3* genes in Arabidopsis, rice, wheat, and cotton also enhanced drought tolerance [17, 64, 84, 89, 91], coinciding with upregulation in both PA16 and PA992 for basic levels of drought tolerance. Our study underscores the pivotal role of heat shock proteins (HSPs) in response to PEG-induced drought stress, particularly in PA992. The strong upregulation of HSPs, including small HSPs and heat stress transcription factors, suggests their crucial function in maintaining cellular integrity under stress. The role of HSPs in drought resilience is well-established, as seen in *GhHSP70-26*, where overexpression in tobacco enhanced drought tolerance, while its silencing in cotton reduced resilience [49]. Similarly, small HSPs have been shown to confer drought tolerance in rice and wheat [59, 79, 80]. Across various plant species, HSP overexpression has been linked to improved drought tolerance by stabilizing proteins and mitigating oxidative stress [49, 59, 78], reinforcing their conserved role in stress adaptation. The differential expression of LEA and HSP genes in PA16 and PA992 suggests that each genotype employs unique yet overlapping molecular strategies to cope with drought stress. While LEA expression appears conserved, HSP induction in PA992 may reflect its adaptive reliance on protein protection pathways under PEG treatment.

### PA992 exhibits enhanced iron homeostasis through differential expression of iron-responsive genes under drought

Drought stress severely impairs the transport of nutrients from root to shoot by reducing the rate of transpiration, which in turn disrupts active ion transport and compromises cell membrane permeability. Maintaining optimal nutrient status is crucial for enhancing plant tolerance and survival under drought conditions [75]. On the other hand, micronutrients like Fe elicit oxidative stress and scavenging Fe may lower oxidative stress. Hence, it would be expected that lower oxidative stress could be associated with increased expression of Fe storage genes in PA16 versus PA992. However, in PA992, the upregulation of several iron-responsive genes under drought stress indicates a robust mechanism for maintaining iron homeostasis, which is critical for mitigating oxidative damage and sustaining metabolic processes. Key genes such as *FRO2*, *NRAMP6*, and *NAS3* were significantly upregulated, reflecting enhanced iron uptake and chelation. Additionally, the elevated expression of *FER2* and *VIT1* suggests an active strategy for iron storage and detoxification, preventing free iron-induced ROS accumulation [9]. The expression of *ISU1*, involved in Fe–S cluster assembly, further highlights the maintenance of mitochondrial function and redox balance in PA992 during drought stress [37]. Together, these responses demonstrate that PA992 activates a coordinated iron homeostasis network, enabling an alternative mechanism to PA16 to better withstand drought-induced oxidative stress. It is thus possible, that the enhanced oxidative stress detected in PA992 as compared with PA16 elicits the Fe homeostasis responses observed, rather than the contrary situation.

Previous studies, including Varshney et al. [77], which provided the draft genome sequence of pigeonpea, and Pahal et al. [51], which reported differential gene expression patterns between pigeonpea genotypes under drought stress, laid essential groundwork for pigeonpea genomics. However, these studies primarily focused on genome annotation and DEG identification. In contrast, our study combines physiological data, transcriptomics, and Weighted Gene Co-expression Network Analysis (WGCNA) to uncover trait-associated gene modules and co-expression networks directly linked to drought tolerance. Moreover, we highlight specific candidate genes such as alpha-farnesene synthase (*AFS*) and LEA proteins, as well as the role of terpenoid biosynthesis, which were not functionally characterized in earlier studies. Thus, our findings provide novel regulatory and functional insights into genotype-specific drought adaptation mechanisms in pigeonpea.

## Conclusion

This study highlights the utility of comparing the drought-tolerant genotype PA16 with the moderately drought-sensitive PA992 to unravel mechanisms underlying drought stress responses in pigeonpea. Transcriptomic analysis under PEG-induced drought stress revealed key regulatory pathways linked to genotype-specific physiological responses. PA16 exhibited reduced oxidative stress (lower H₂O₂ and TBARS levels) and higher proline content, alongside the upregulation of genes involved in terpenoid and flavonoid biosynthesis, contributing to enhanced drought resilience. In contrast, PA992 showed higher ROS accumulation and activated genes related to iron homeostasis, reflecting a moderate and more reactive stress response. Despite their differences, both genotypes exhibited induction of common drought-responsive genes, including heat shock proteins (HSPs), Late Embryogenesis Abundant (LEA) proteins, and dehydrins, which serve as central players in stress protection. The graphical summary (Fig. S6) illustrates these genotype-specific and shared responses, emphasizing how coordinated transcriptional adjustments contribute to drought tolerance.

## Future prospects

To build on these findings, functional validation of candidate regulatory genes—such as alpha-farnesene synthase (AFS) and dehydrins (LEA3, XERO1)—should be prioritized using approaches like CRISPR-Cas9 or RNAi. Additionally, targeted metabolomics studies, particularly LC–MS/MS using curated reference databases, are recommended to profile drought-induced terpenoids and explore their functional significance. These integrated transcriptomic and metabolomic efforts will further elucidate the biochemical pathways contributing to drought resilience in pigeonpea.

## Supplementary Information

Additional file 1: Figure S1- (a) Heatmap displaying differentially expressed genes (DEGs) responsive to PEG-induced drought stress in Cajanus cajan genotypes PA16 and PA992 under treated and control conditions. The color gradient represents log₂ fold-change values, transitioning from blue (downregulation) to yellow (upregulation), with intermediate shades indicating moderate expression changes. (b) Correlation analysis of drought-responsive DEGs from PA16 and PA992 with key WGCNA modules (turquoise, brown, and yellow), highlighting their module membership under drought stress conditions

Additional file 2: Figure S2- (a) Co-expression network of the DEGs indicative of secondary metabolism. The lines illustrate gene interactions in Cytoscape, with node size and the color gradient signifying the combined score. (b) The illustration represents a hypothetical model of mevalonate and 2-C-methyl-D-erythritol-4-phosphate pathway. On the left side of the figure, genes encoding regulatory responsive enzymes for terpenoid, and flavanol synthesis are listed in the box. Here, AACT: Acetyl CoA C- Acetyltransferase, HMG(S): Hydroxymethylglutaryl-CoA (synthase), HMGR: Hydroxymethylglutaryl-CoA reductase, G3P: glyceraldehyde-3-phosphate, DXS: 1-deoxy-D-xylulose-5-phosphate synthase, DXP: 1-deoxy-D-xylulose-5-phosphate, IPP: isopentenyl diphosphate, DMAPP: dimethylallyl diphosphate, FPP: farnesyl pyrophosphate, TPS: Terpene synthase, NPP: neryl diphosphate, GPP: geranyl diphosphate, CHS: Chalcone synthase, G8H: geraniol 8-hydroxylase, MAS: momilactone A synthase, NES: nerolidol synthase, AFS: α-farnesene synthase. Figure has been created with the help of https://BioRender.com

Additional file 3: Figure S3- The heatmap illustrates KEGG pathways linked to differentially expressed genes, with red highlighting pathways enriched with upregulated DEGs and green denoting those with downregulated DEGs. GAGE analysis was performed, and the most significant pathways were visualized using the Pathview web tool. The color bar represents the intensity and log2 fold change values of the DEGs. The gradient transitions from blue (arrow in downward direction), representing downregulation, to red, indicating upregulation (arrow in upward direction)

Additional file 4: Figure S4- Heatmap of the DEGs responsive to iron homeostasis in PA16, PA992, treated, and control conditions under PEG-induced drought stress. The color bar reflect log2-fold change values. The gradient transitions from blue to yellow, indicating down and upregulation respectively, with intermediate colors representing gradual changes in fold change values

Additional file 5: Figure S5- Validation of gene regulation by RT-qPCR. Representative gene expression, as indicated, showing the validation of selected DEGs. The error bar signifies the standard deviation of the mean, calculated using the geomean of 3 replicates. The responses are compared in PA16 and PA992 under PEG-induced drought stress (PEG) versus the control (ctrl) (Table S6). The asterisk (*) denotes the significant difference between the control and treated conditions calculated using the unpaired t-test in the GraphPad Prism. Significant codes with respect to *p*-values are *** 0.001, ** 0.01, and * 0.05.

Additional file 6: Figure S6- Overview of genotype-specific drought stress responses in pigeonpea. The drought-tolerant genotype Pusa Arhar 16 (PA16) shows reduced H₂O₂ and TBARS levels, increased proline accumulation, and activation of terpenoid and flavonoid biosynthesis genes, resulting in enhanced drought resilience. In contrast, the drought-sensitive Pusa 992 displays elevated ROS and TBARS, reduced proline, and upregulation of iron homeostasis-related genes, reflecting a moderate drought response. Both genotypes exhibit upregulation of shared stress-responsive genes including HSPs, LEA proteins, and dehydrins, indicating core drought defense mechanisms

Additional file 7: Table S1- RNA-Seq Read Mapping Summary. Summary of RNA-Seq data for control and treated samples of PA16 and PA992, each in triplicate. The table presents the total number of reads generated and the percentage of uniquely mapped reads aligned to the pigeonpea genome, providing an overview of sequencing quality and mapping efficiency

Additional file 8: Table S2- Differentially expressed genes in response to drought stress. It contains a mother table, filtered DEGs with *p*-value ≤ 0.05 & log2fold changes less than -1 and greater than 1, lists of up and down-regulated genes under PA16 and PA992 (PEG vs. ctrl), and PEG and ctrl (PA16 vs. PA992).

Additional file 9: Table S3- Weighted gene correlation network analysis (WGCNA) Module-Based Grouping of Differentially Expressed Genes. List of differentially expressed genes (DEGs) grouped into co-expression modules identified by Weighted Gene Co-expression Network Analysis (WGCNA). Each module represents a set of genes with similar expression patterns, potentially involved in specific biological processes related to stress response in PA16 and PA992

Additional file 10: Table S4- This table includes DEGs related to hormones, transcription factors, secondary metabolites, LEA proteins, heat shock proteins (HSPs), and drought-responsive genes and its related DEGs linked to WGCNA modules and genes involved in iron homeostasis, compared to their respective controls

Additional file 11: Table S5- Gene ontology (GO) terms related to biological processes and molecular functions in all samples. The listed GO terms are associated with differentially expressed genes (DEGs) in PA16 and PA992 under PEG vs. control conditions, as well as in PEG-treated PA16 vs. PA992. The table includes GO terms linked to both upregulated and downregulated genes, providing insights into their functional roles in stress adaptation

Additional file 12: Table S6- List of Primers for qRT-PCR. The qRT-PCR was performed for function validation of some randomly selected genes in PEG-induced drought stress in PA16 and PA992

Additional file 13: Table S7. Summary of Gene Ontology (GO) terms enriched in WGCNA-derived modules and their functional associations with physiological traits under drought stress in pigeonpea. This table lists the major biological functions (GO terms) associated with genes within each module, and further outlines their inferred role in modulating key physiological traits such as relative water content (RWC), proline accumulation, and oxidative stress markers (MDA, H₂O₂). The associations are based on GO enrichment analysis and trait-specific trends observed in PA16 and PA992 genotypes under drought conditions

## Data Availability

Data Availability Statement The original contributions presented in the study are publicly available. This data can be found here: Bioproject: PRJNA1250142 (BioSample: SAMN47931845, SAMN47931846, SAMN47931847, and SAMN47931848).
