# Supplementary material for: Transcriptional regulation reveals potent drought tolerance mechanisms in contrasting genotypes of Cajanus cajan (L.) Millspaugh
Source: BMC Plant Biol. 2025 Oct 2;25:1287. doi: 10.1186/s12870-025-07174-6 (PMC12490149; doi:10.1186/s12870-025-07174-6)
Supplement: Supplementary file 1 — Additional file 1: Figure S1- (a) Heatmap displaying differentially expressed genes (DEGs) responsive to PEG-induced drought stress in Cajanus cajan genotypes PA16 and PA992 under treated and control conditions. The color gradient represents log₂ fold-change values, transitioning from blue (downregulation) to yellow (upregulation), with intermediate shades indicating moderate expression changes. (b) Correlation analysis of drought-responsive DEGs from PA16 and PA992 with key WGCNA modules (turquoise, brown, and yellow), highlighting their module membership under drought stress conditions [file 12870_2025_7174_MOESM1_ESM.pdf]

Drought Responsive Genes

(a)

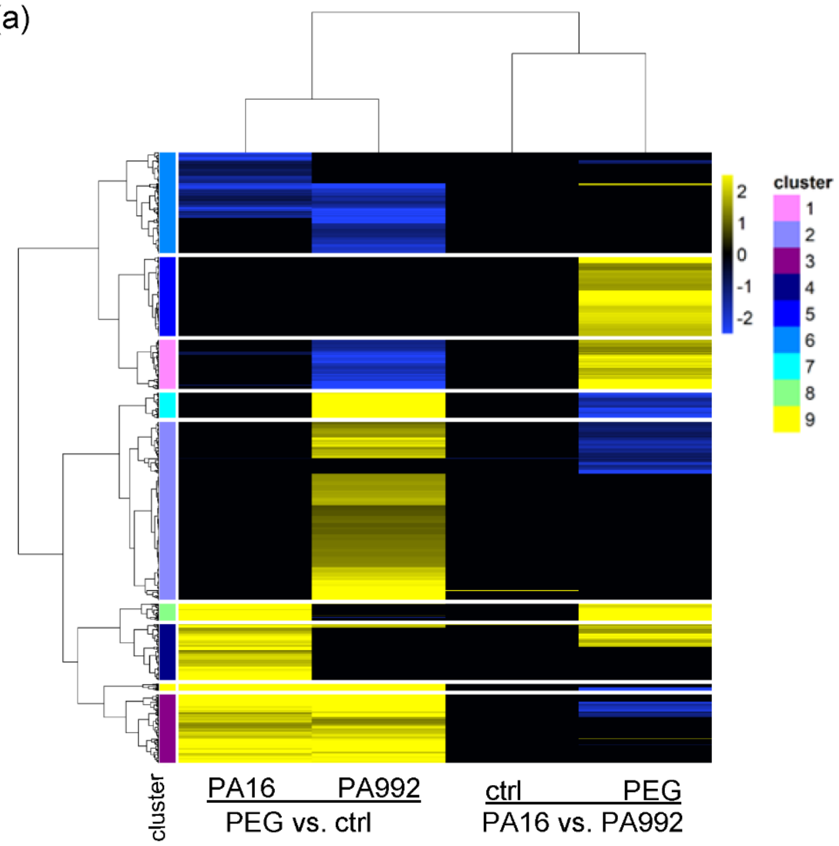


(b) Drought Response Correlation Between PA16 and PA992 Varieties

Pearson R= 0.935

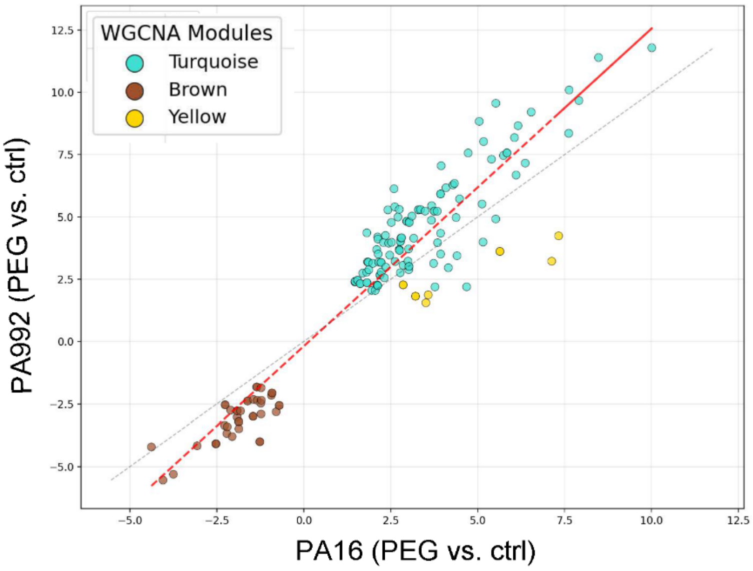

Figure S1- (a) Heatmap displaying differentially expressed genes (DEGs) responsive to PEG-induced drought stress in Cajanus cajan genotypes PA16 and PA992 under treated and control conditions. The color gradient represents $\log_2$ fold-change values, transitioning from blue (downregulation) to yellow (upregulation), with intermediate shades indicating moderate expression changes. (b) Correlation analysis of drought-responsive DEGs from PA16 and PA992 with key WGCNA modules (turquoise, brown, and yellow), highlighting their module membership under drought stress conditions.
