# Supplementary material for: Transcriptional regulation reveals potent drought tolerance mechanisms in contrasting genotypes of Cajanus cajan (L.) Millspaugh
Source: BMC Plant Biol. 2025 Oct 2;25:1287. doi: 10.1186/s12870-025-07174-6 (PMC12490149; doi:10.1186/s12870-025-07174-6)
Supplement: Supplementary file 2 — Additional file 2: Figure S2- (a) Co-expression network of the DEGs indicative of secondary metabolism. The lines illustrate gene interactions in Cytoscape, with node size and the color gradient signifying the combined score. (b) The illustration represents a hypothetical model of mevalonate and 2-C-methyl-D-erythritol-4-phosphate pathway. On the left side of the figure, genes encoding regulatory responsive enzymes for terpenoid, and flavanol synthesis are listed in the box. Here, AACT: Acetyl CoA C- Acetyltransferase, HMG(S): Hydroxymethylglutaryl-CoA (synthase), HMGR: Hydroxymethylglutaryl-CoA reductase, G3P: glyceraldehyde-3-phosphate, DXS: 1-deoxy-D-xylulose-5-phosphate synthase, DXP: 1-deoxy-D-xylulose-5-phosphate, IPP: isopentenyl diphosphate, DMAPP: dimethylallyl diphosphate, FPP: farnesyl pyrophosphate, TPS: Terpene synthase, NPP: neryl diphosphate, GPP: geranyl diphosphate, CHS: Chalcone synthase, G8H: geraniol 8-hydroxylase, MAS: momilactone A synthase, NES: nerolidol synthase, AFS: α-farnesene synthase. Figure has been created with the help of https://BioRender.com [file 12870_2025_7174_MOESM2_ESM.pdf]

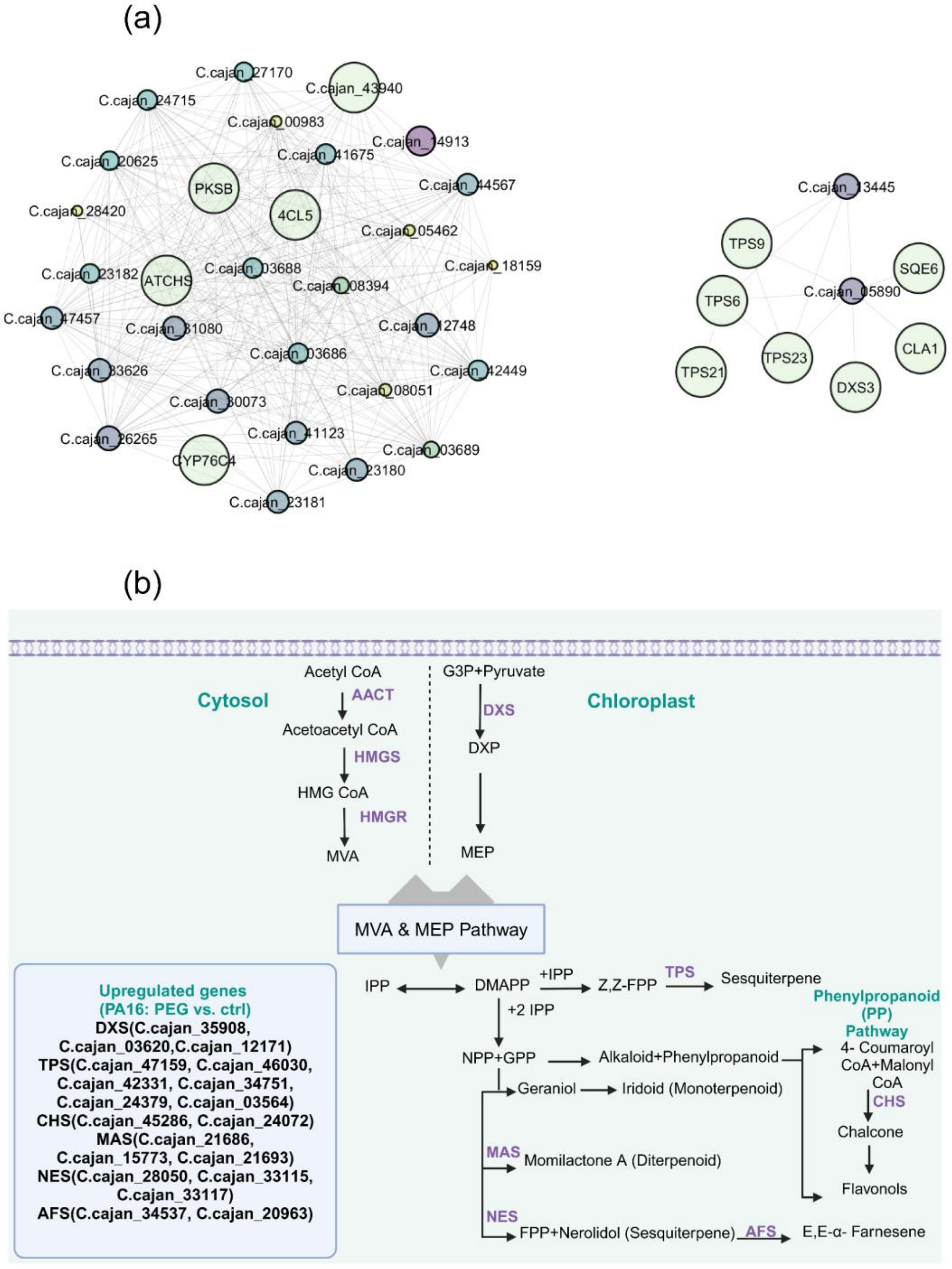


Figure S2- (a) Co-expression network of the DEGs indicative of secondary metabolism. The lines illustrate gene interactions in Cytoscape, with node size and the color gradient signifying the combined score. (b) The illustration represents a hypothetical model of mevalonate and 2-C-methyl-D-erythritol-4-phosphate pathway. On the left side of the figure, genes encoding regulatory responsive enzymes for terpenoid, and flavanol synthesis are listed in the box. Here, AACT: Acetyl CoA C-

Acetyltransferase, HMG(S): Hydroxymethylglutaryl-CoA (synthase), HMGR: Hydroxymethylglutaryl-CoA reductase, G3P: glyceraldehyde-3-phosphate, DXS: 1-deoxy-D-xylulose-5-phosphate synthase, DXP: 1-deoxy-D-xylulose-5-phosphate, IPP: isopentenyl diphosphate, DMAPP: dimethylallyl diphosphate, FPP: farnesyl pyrophosphate, TPS: Terpene synthase, NPP: neryl diphosphate, GPP: geranyl diphosphate, CHS: Chalcone synthase, G8H: geraniol 8-hydroxylase, MAS: momilactone A synthase, NES: nerolidol synthase, AFS: α-farnesene synthase. Figure has been created with the help of https://BioRender.com.
