# Supplementary material for: Transcriptional regulation reveals potent drought tolerance mechanisms in contrasting genotypes of Cajanus cajan (L.) Millspaugh
Source: BMC Plant Biol. 2025 Oct 2;25:1287. doi: 10.1186/s12870-025-07174-6 (PMC12490149; doi:10.1186/s12870-025-07174-6)
Supplement: Supplementary file 3 — Additional file 3: Figure S3- The heatmap illustrates KEGG pathways linked to differentially expressed genes, with red highlighting pathways enriched with upregulated DEGs and green denoting those with downregulated DEGs. GAGE analysis was performed, and the most significant pathways were visualized using the Pathview web tool. The color bar represents the intensity and log2 fold change values of the DEGs. The gradient transitions from blue (arrow in downward direction), representing downregulation, to red, indicating upregulation (arrow in upward direction) [file 12870_2025_7174_MOESM3_ESM.pdf]

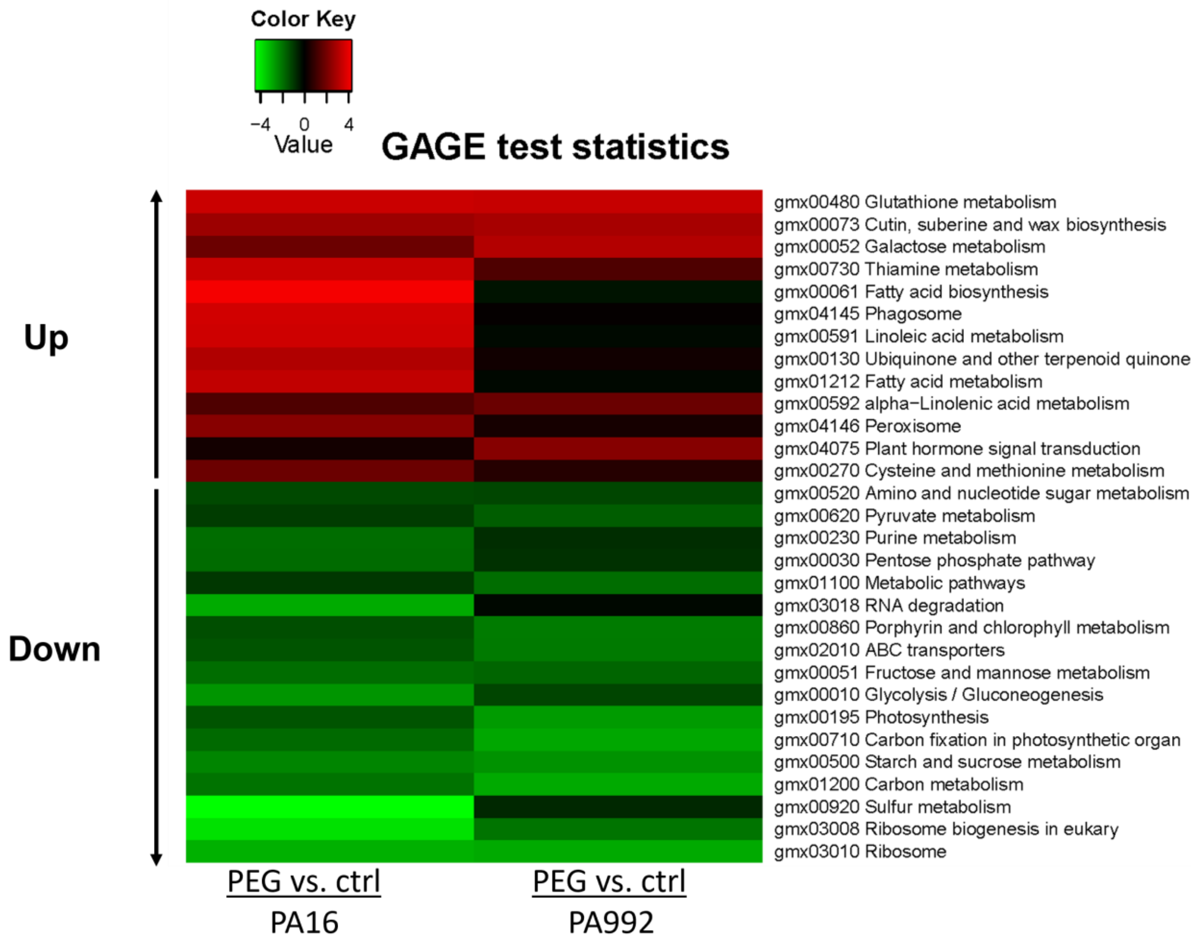

Figure S3- The heatmap illustrates KEGG pathways linked to differentially expressed genes, with red highlighting pathways enriched with upregulated DEGs and green denoting those with downregulated DEGs. GAGE analysis was performed, and the most significant pathways were visualized using the Pathview web tool. The color bar represents the intensity and log2 fold change values of the DEGs. The gradient transitions from blue (arrow in downward direction), representing downregulation, to red, indicating upregulation (arrow in upward direction).
