# Supplementary material for: Transcriptional regulation reveals potent drought tolerance mechanisms in contrasting genotypes of Cajanus cajan (L.) Millspaugh
Source: BMC Plant Biol. 2025 Oct 2;25:1287. doi: 10.1186/s12870-025-07174-6 (PMC12490149; doi:10.1186/s12870-025-07174-6)
Supplement: Supplementary file 4 — Additional file 4: Figure S4- Heatmap of the DEGs responsive to iron homeostasis in PA16, PA992, treated, and control conditions under PEG-induced drought stress. The color bar reflect log2-fold change values. The gradient transitions from blue to yellow, indicating down and upregulation respectively, with intermediate colors representing gradual changes in fold change values [file 12870_2025_7174_MOESM4_ESM.pdf]

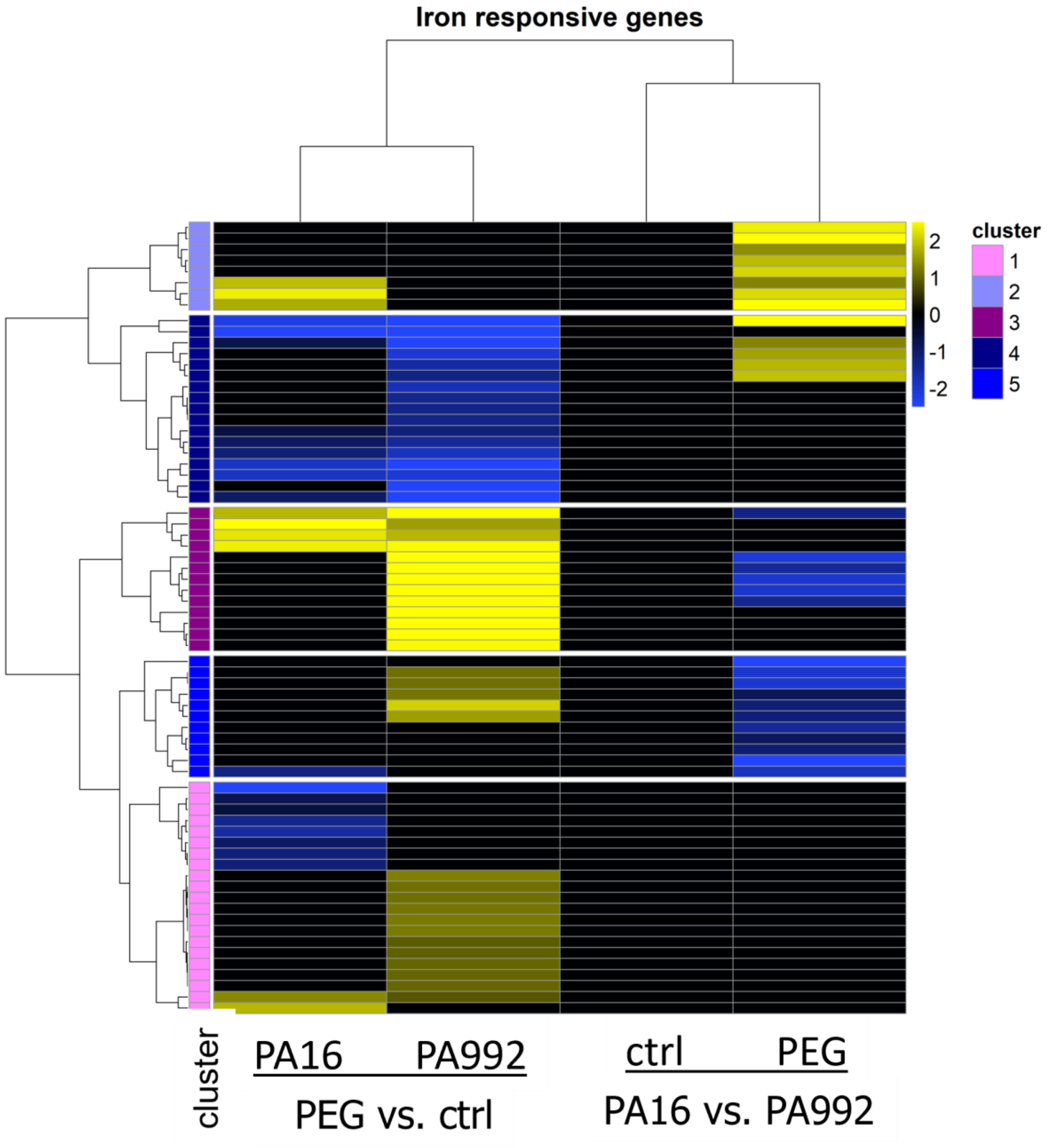

Figure S4- Heatmap of the DEGs responsive to iron homeostasis in PA16, PA992, treated, and control conditions under PEG-induced drought stress. The color bar reflect log2-fold change values. The gradient transitions from blue to yellow, indicating down and upregulation respectively, with intermediate colors representing gradual changes in fold change values.
