# Supplementary material for: Transcriptional regulation reveals potent drought tolerance mechanisms in contrasting genotypes of Cajanus cajan (L.) Millspaugh
Source: BMC Plant Biol. 2025 Oct 2;25:1287. doi: 10.1186/s12870-025-07174-6 (PMC12490149; doi:10.1186/s12870-025-07174-6)
Supplement: Supplementary file 5 — Additional file 5: Figure S5- Validation of gene regulation by RT-qPCR. Representative gene expression, as indicated, showing the validation of selected DEGs. The error bar signifies the standard deviation of the mean, calculated using the geomean of 3 replicates. The responses are compared in PA16 and PA992 under PEG-induced drought stress (PEG) versus the control (ctrl) (Table S6). The asterisk (*) denotes the significant difference between the control and treated conditions calculated using the unpaired t-test in the GraphPad Prism. Significant codes with respect to p-values are *** 0.001, ** 0.01, and * 0.05. [file 12870_2025_7174_MOESM5_ESM.pdf]

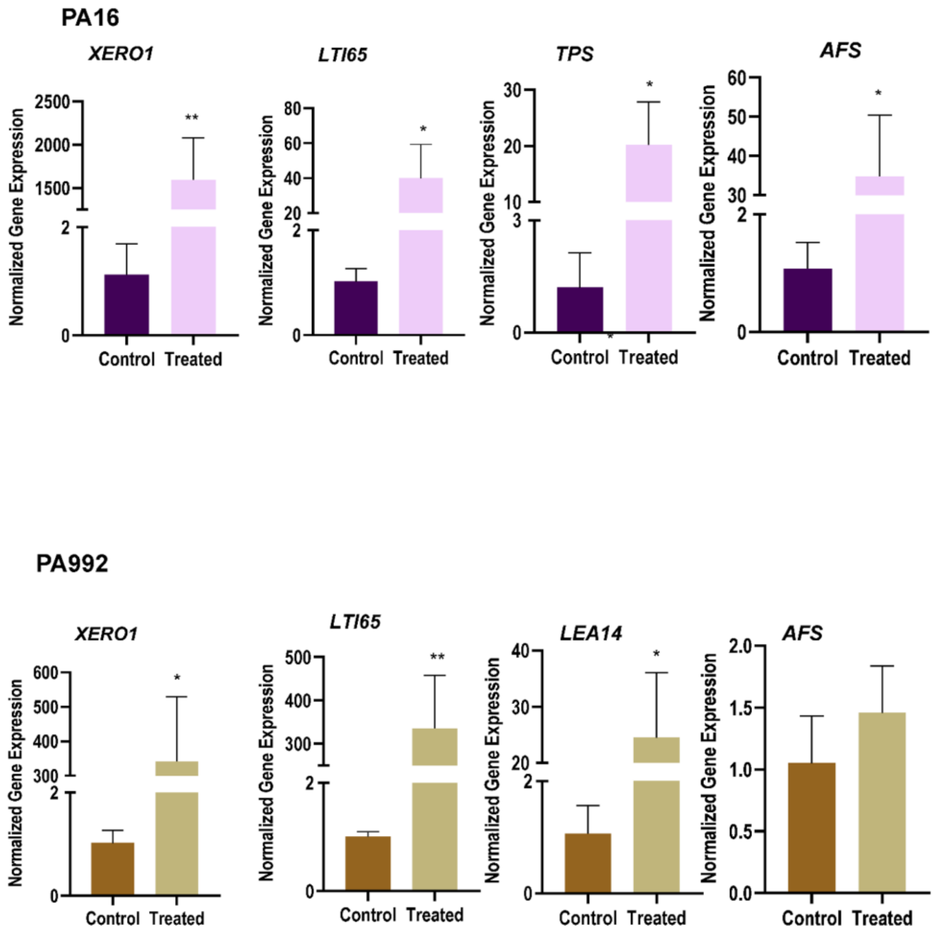

Figure S5- Validation of gene regulation by RT-qPCR. Representative gene expression, as indicated, showing the validation of selected DEGs. The error bar signifies the standard deviation of the mean, calculated using the geomean of 3 replicates. The responses are compared in PA16 and PA992 under PEG-induced drought stress (PEG) versus the control (ctrl) (Table S6). The asterisk (*) denotes the significant difference between the control and treated conditions calculated using the unpaired t-test in the GraphPad Prism. Significant codes with respect to p-values are *** 0.001, ** 0.01, and * 0.05.
