# Supplementary material for: Transcriptional regulation reveals potent drought tolerance mechanisms in contrasting genotypes of Cajanus cajan (L.) Millspaugh
Source: BMC Plant Biol. 2025 Oct 2;25:1287. doi: 10.1186/s12870-025-07174-6 (PMC12490149; doi:10.1186/s12870-025-07174-6)
Supplement: Supplementary file 6 — Additional file 6: Figure S6- Overview of genotype-specific drought stress responses in pigeonpea. The drought-tolerant genotype Pusa Arhar 16 (PA16) shows reduced H₂O₂ and TBARS levels, increased proline accumulation, and activation of terpenoid and flavonoid biosynthesis genes, resulting in enhanced drought resilience. In contrast, the drought-sensitive Pusa 992 displays elevated ROS and TBARS, reduced proline, and upregulation of iron homeostasis-related genes, reflecting a moderate drought response. Both genotypes exhibit upregulation of shared stress-responsive genes including HSPs, LEA proteins, and dehydrins, indicating core drought defense mechanisms [file 12870_2025_7174_MOESM6_ESM.pdf]

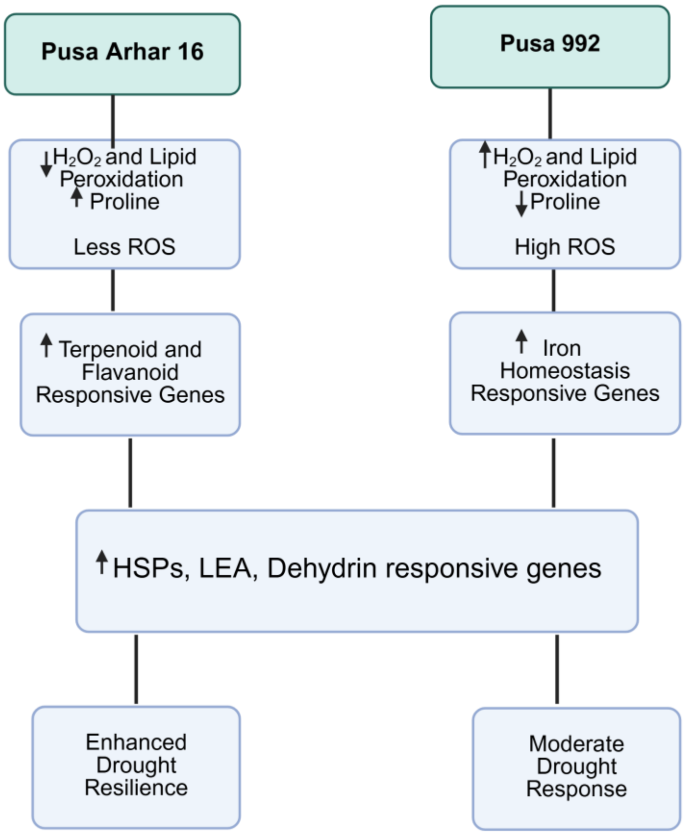

Figure S6- Overview of genotype-specific drought stress responses in pigeonpea. The drought-tolerant genotype Pusa Arhar 16 (PA16) shows reduced H_2O_2 and TBARS levels, increased proline accumulation, and activation of terpenoid and flavonoid biosynthesis genes, resulting in enhanced drought resilience. In contrast, the drought-sensitive Pusa 992 displays elevated ROS and TBARS, reduced proline, and upregulation of iron homeostasis-related genes, reflecting a moderate drought response. Both genotypes exhibit upregulation of shared stress-responsive genes including HSPs, LEA proteins, and dehydrins, indicating core drought defense mechanisms.
